# TriCAM (NCT02976558) – a randomized controlled pilot study of complementary medicine in allogeneic stem cell transplantation to improve quality of life

**DOI:** 10.1186/s12906-025-05058-8

**Published:** 2025-09-08

**Authors:** Ali Behzad, Stefan W. Krause, Andreas Mackensen, Fabian Müller, Anja Stark, Simon Völkl, Wolf Rösler

**Affiliations:** 1 Department of Internal Medicine 5 – Hematology/Oncology, University Hospital of Erlangen, Erlangen, Germany; 2 Department for Internal and Integrative Medicine, Sozialstiftung Bamberg, Bamberg, Germany

**Keywords:** Allogeneic hematopoietic stem cell transplantation, Complementary medicine, Acupuncture, Clown therapy, Music therapy, Quality of life, Randomized controlled pilot trial

## Abstract

**Background:**

Allogeneic stem cell transplantation (aSCT) frequently leads to prolonged impaired quality of life (Qol) and depression. To reduce symptom burden and improve Qol, we implemented a complementary medicine approach (TriCAM).

**Methods:**

In a randomized, controlled clinical trial (NCT02976558), we enrolled 36 patients undergoing aSCT. In addition to standard care, the intervention group received Traditional Chinese Medicine acupuncture (TCMA), music therapy (TaKeTiNa) and active clown role playing. FACT-G was used to assess Qol and HADS-D to asses anxiety and depression. We monitored safety of TCMA as well as typical complications during aSCT.

**Results:**

TCMA did not result in more hematomas, soft tissue infections or bacteriaema. Clinical depression was reduced in the intervention group compared to the control group (*p* = 0.01). There was a trend towards improvement of Qol over time (*P* = 0.10) and in reducing aGvHD (*P* = 0.084) in the intervention group compared to the control group.

**Conclusions:**

Our trial is the first to show TCMA to be safe in aSCT. TriCAM showed a trend towards mitigating the loss of QoL and significantly reduced depression in patients undergoing aSCT. The promising results of this pilot study give a first positive signal for safety and efficacy allowing for the design of larger studies to confirm the results and support this particularly vulnerable patient group.

**Trial registration:**

ClinicalTrials.gov NCT02976558. Registered 29 November 2016.

**Supplementary Information:**

The online version contains supplementary material available at 10.1186/s12906-025-05058-8.

## Background

Allogeneic stem cell transplantation (aSCT) is the only curative option for several hematological diseases such as acute myeloid leukemia, depending on individual disease risk [1]. During the course of the transplantation, nearly 50% of these patients develop acute Graft versus Host Disease (aGvHD) [2]. Apart from substantial treatment related mortality (TRM) [3], patients receiving aSCT can also suffer substantial and long-term side effects, compromising daily life activity and quality of life (Qol) [4]. After transplantation, over 30% of patients develop depression or posttraumatic stress disease (PTSD) despite psycho-oncological interventions [5]. Aside from decreased Qol, overall survival is lower in such distressed patients [6]. In the years from 2012–2014, our team looked into additional therapeutic approaches that could help to mitigate PTSD, depression and improve Qol of the patients. In addition to standard care psycho-oncological support, we started out to test complimentary methods in individual patients who had developed major depressions or were severely sick. From the vast number of therapies used, the three approaches that were reported consistently by patients to be effective were further investigated from 2014–2016 in a multimodal approach: Traditional Chinese Medicine Acupuncture (TCMA) [7], TaKeTiNa®-music-therapy [8] and Metzler®-clown-roleplaying [9]. With a lack of known evidence-based therapies to improve Qol and mitigate depression, we decided to investigate them in a randomized controlled clinical trial (RCT). We present our findings. TCM view on diagnosis, disease development and treatment will be presented elsewhere.

## Methods

We performed RCT pilot study (clinicaltrials.gov: NCT02976558, first registration 29.11.2016). The recruitment period was January 2017 to March 2019 and we planned to recruit 104 patients according to the sample size calculation described below. After informed consent, we enrolled a total of 36 patients undergoing aSCT at the University Hospital of Erlangen. Despite generally frequent inclusion of patients and high acceptance rates of 95%, the study was prematurely closed due to logistical challenges, including the departure of key study personnel (e.g., the primary acupuncturist and study nurse) and insufficient funds to continue recruitment beyond 36 patients. Inclusion criteria were age ≥ 18 years regardless of their hematological malignancies and their remission state before transplantation. Exclusion criteria were start of conditioning chemotherapy before inclusion, active participation in other clinical trials and clinical depression defined by a Hospital Anxiety and Depression Score Germany (HADS-D Score) of ≥ 11 and antidepressant drug treatment. At inclusion, patients were given the questionnaires of the first visit before knowing which group they were in to reduce the influence of potential disappointment into the first dataset.

### Control group and blinding

Since the interventions as a whole could not be placebo-controlled or blinded, the control group received treatment as usual undergoing allogeneic stem cell transplantation, which in our department includes periodical psycho-oncological guidance. Questionnaires and outcome parameters mentioned below were collected and documented by our GCP certified study center for both groups equally. Once randomized, patients in the control group were offered a free one-time consultation on CAM after the study period.

### Randomization

The random allocation sequence was generated in one block with a block size of 4 using randomization.com. The results were sealed in a non-see-through envelope that was randomly mixed within the block. After enrollment in the study, the study nurse was informed. An independent person that was assigned for the study then drew one of the letters, opened it, wrote it on a list with signature and date. The study nurse then wrote the date of the randomization and the result into the electronic documentation system (eCRF). Study participants that didn´t finish the study were not replaced.

### Intervention

**Fig. 1:**
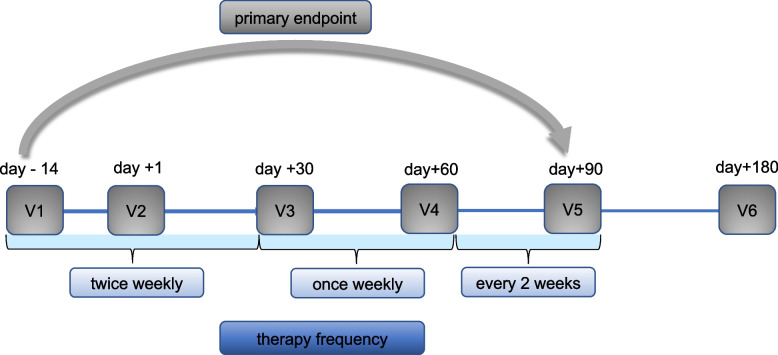
Study design and trial profile. The figure shows the six clinical visits and their timeline (V1-V6). The intervention group received the multimodal therapy consisting of 20 min of TCMA and 10 min of each TaKeTiNa and Clown-therapy. The therapy was received twice weekly in the first month, then once weekly the 2nd month and once every two weeks for the 3rd month

All participants randomized in the intervention group were offered a multimodal therapy of complementary medicine always consisting of three methods e.g., TCMA, music therapy, and clown therapy between the visits at days −14, 0, 30, 60 and 90 as outlined in Fig. 1. TCMA was performed by a board-certified acupuncturist. TaKeTiNa sessions were led by a certified TaKeTiNa therapist. Clown therapy was conducted by a medical doctor trained in the Metzler method. Therapist-led interventions were delivered independently of each other and study visits. Each was scheduled twice weekly in the first month, weekly in the second, and biweekly in the third. Post-discharge, interventions aligned with study visits. Patients were advised to practice TaKeTiNa and clown exercises independently between sessions using a booklet, though frequency was unspecified and adherence was not monitored. There were no specific clinical criteria required for their application. The 10-min duration for TaKeTiNa and clown therapy was chosen based on our prior clinical experience (2014–2016) with individual aSCT patients, which indicated that shorter sessions were better tolerated amid treatment-related symptoms and fatigue while still reported as beneficial. TCMA: Patients were diagnosed according to TCM by a board-certified acupuncturist using the 5 elements, the 8 principles and pathogenic factors on each visit according to Kaptchuk 2002 [10]. Pulse and tongue diagnosis were performed at each visit. Before every acupuncture session, a brief assessment was made using the above diagnostic tools. Then, depending on the underlying current imbalance, 5 to 20 acupuncture points were selected. After skin disinfection, needles were inserted until a DeQi sensation (a feeling of spreading warmth or electric sensation) was reported by the patient. Needles with a 0.16 mm diameter and 15 mm length (Seirin® B type needle No.1, Japan) were used to minimize the risk of bleeding or infections. Unless discomfort was reported by the patient or a change in position was necessary, in which case the needles were removed, the needles remained for a total of 10–20 min before being removed. Patients with minor bleeding complications (e.g., small hematomas) remained eligible for TCMA, provided needles were not inserted into affected areas or sites with high bleeding or infection risk (e.g., thrombosis). No patients experienced severe bleeding complications that contraindicated acupuncture. Other than that, acupuncture was performed irrespective of fever, systemic inflammation, aGvHD, neutropenia or thrombocytopenia.

#### TaKeTiNa music therapy

During the first intervention, the principles of TaKeTiNa® were explained to the patient by a certified TaKeTiNa® therapist. Egg-shakers (NINOSET540®, Meinl, Germany) were used as percussion instruments. An additional booklet containing further instructions was given to the patients and they were encouraged to do exercises on their own in between the interventions (suppl. mat. 1: TriCAM Exercise Booklet) Then and in subsequent therapy-sessions lasting about 10 min, a one-on-one TaKeTiNa®-session was conducted laying, sitting or standing depending on the patients’ condition and preference using voice, feet, hands and optionally egg-shakers.

#### Metzler®-Clown-Therapy

During the first intervention, the principles of Metzler-Clown-Therapy [9] were explained to the patient by a medical doctor with the necessary training. A clown nose was given to the patient as the smallest form of masquerading. Patients were encouraged to perform exercises from the booklet mentioned above on their own between the interventions. Then and in subsequent therapy-sessions lasting about 10 min, exercises were conducted laying, sitting or standing depending on the patients’ condition and preference using the 6 principles of relationship, roleplaying and archetypical characters (“Kellerkinder”).

### Primary and secondary outcomes

The follow-up period for primary and all secondary outcomes was 6 months post-transplantation (visit 6). Data regarding the outcome parameters onset of GvHD, overall survival (OS), progression-free survival (PFS) and treatment related mortality (TRM) was assessed for up to 1 year of follow-up.

#### FACT-G

The Functional Assessment of Cancer Therapy (German version) was used to assess Qol. The primary endpoint of the study was the difference in the mean change of the FACT-G total score visit 5 compared to visit 1 between the groups.

#### Secondary endpoints: HADS-D

To evaluate the depression and anxiety of the patients, the Hospital Anxiety and Depression Score (German version; HADS-D) was used. A secondary endpoint was the mean change of the HADS-D depression and HADS-D anxiety score visit 5 compared to visit 1 between the groups.

FACT-G and HADS-D questionnaires were completed at each study visit (V1–V6) to assess QoL, depression, and anxiety over time, with the primary endpoint analysis focusing on the change from visit 1 to visit 5. Higher FACT-G scores indicate better QoL, while higher HADS-D scores reflect greater levels of anxiety or depression.

#### Safety of acupuncture in aSCT

Since the patients were highly immunosuppressed and had temporary severe thrombocytopenia, we compared the cumulative incidence of soft-tissue-hematomas or soft-tissue-infections requiring treatment, defined as topical (e.g. band-aids) or systemic (e.g. antibiotics) between the groups from visit 1 to visit 5.

Other endpoints at month 3 (visit 5) included ECOG Performance Status, Karnofsky Performance Status, pneumonias, viral infections, hospitalization days, and stationary costs from visit 1 through visit 5 between the groups.

### Statistical analysis

Due to the lack of previous scientific data, we calculated the effect size of the primary outcome parameter after 5 patients of each group had been enrolled and had completed visit 5 using a two-sided Wilcoxon-Mann–Whitney test. From this estimated effect size, a target population of 104 patients was calculated. The analysis was planned as intention-to-treat (ITT), so that from visit 2 on, in case of a premature dropout before visit 5 (endpoint), the last documented visit was used as visit 5 for that patient. We planned prospectively to measure the differences in groups for endpoints FACT-G, HADS-D by performing a two-sided WMW-Test. To assess the development of FACT-G sum-score and subgroups over time (V1–V6), a linear mixed-effects model was used, the mixed effect ANOVA, which incorporates fixed effects for group (intervention vs. control), visit (time), and their interaction (group * visit) and a random effect for patients to account for repeated measures. The *P*-value for the group * visit interaction indicates whether the QoL trajectory differed between groups. As the found interaction was not significant, pairwise comparisons between groups were omitted. The survival probabilities (OS, PFS) and incidence of aGvHD were estimated by using the Kaplan‐Meier method and were compared using the log‐rank test. For other endpoints shown supp. table 1- 3, two-sided p-test was performed. If not otherwise stated, error bars in the graphs display the mean SD. The results were generated by Graphpad-Prism® version 9.1.2

## Results

### Patient demographics and clinical characteristics

**Fig. 2:**
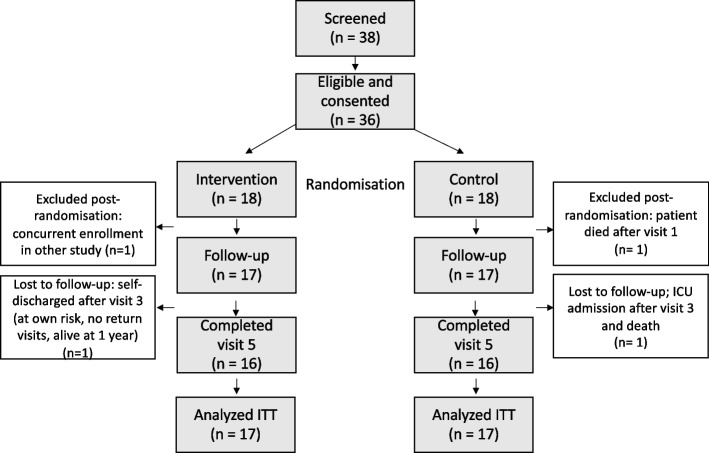
Participant flow following the CONSORT guidelines. ITT = intention to treat

Between January 2017 and March 2019, we recruited 36 patients into the study, as seen in the flow chart (Fig. 2). The interest of putative participants in the intervention was very high (95% of patients approached). One patient in the intervention group was excluded due to participation in another clinical study (screening failure), another patient in the control group died due to complications short after the first visit and was also excluded. From the 34 patients, one patient in the intervention group left the hospital at own risk after visit 3 and was still alive one year later, another patient in the control group was admitted to ICU after visit 3 on and died there. 32 patients completed visit 5. Table 1 shows the patients characteristics.

**Table 1:** Participant characteristics

Characteristics	Control	Intervention	*P*-value
Age in years, median (range)	58 (24–73)	57 (26–71)	0.82
Sex, n (%)			0.17
Male	10 (58.8)	6(35.3)	
Female	7 (41.2)	11 (64.7)	
Sorror Score average	1.53	1.47	0.71
Underlying disease, n			0.63
Acute myelogenous leukemia (AML)	6	5	
Acute lymphocytic leukemia (ALL)	1	3	
Non-Hodgkin’s lymphoma	1	0	
Myelodysplastic syndromes (MDS)	6	3	
Multiple myeloma	1	1	
MPN (incl. CML)	2	5	
Diease Risk Index^*^ (average)	2.2	2.7	0.15
Low	1	0	
Intermediate	12	9	
High	4	5	
Very high	0	3	
Conditioning regimen, n (%)			
Myeloablative	9 (52.9)	9 (52.9)	1
Nonmyeloablative	8 (47.1)	8(47.1)	1
Total body Iradiation (TBI) > 6 Gy	1 (5.9)	2 (11.8)	1
Antithymocyte globulin use, n (%)	17 (100)	13 (76.5)	0.1
Graft source, n (%)			N/A
Peripheral blood	17 (100)	17 (100)	
Donor type, n (%)			0.07
Haploidentical	0 (0)	4 (23.5)	
Matched related	1 (5,9)	2 (11.8)	
Matched unrelated	14 (82.3)	8 (47.1)	
Mismatched	2 (11.8)	3 (17.7)	
Second transplantation	0 (0)	2 (11.8)	0.48
Female donor and male recipient	2 (11.8)	0 (0)	0.48
GvHD Prophylaxis, n (%)			0.1
Ciclosporin + Methotrexate + MMF	15 (82)	10 (47)	
Tacrolimus + Methotrexate + MMF	2 (11.8)	3 (17.7)	
PT-Cyclophosphamid + Tacrolimus + MMF	0 (0)	4 (24)	

^*^Diease risk index from CIBMTR 2019

### Quality of life using FACT-G

**Fig. 3:**
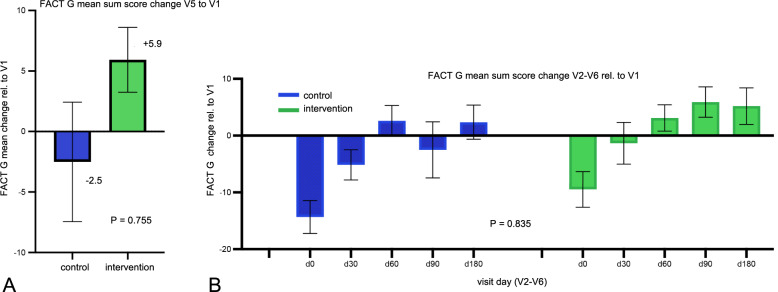
**a** (left): Difference of the mean Fact-G sum score V5 to V1 according to treatment group. The higher the value, the higher the Qol; **b** (right): The graph shows the mean change from baseline FACT-G sum score to the different visits in each group. Visit 2 is the day after transplantation, visit 3 one month after transplantation, visit 4 two months, visit 5 three months after transplantation. For visual purposes, error bars show the mean with SEM

A total of 16 patients in the control group and 16 patients in the intervention group underwent visit 5 FACT-G assessments (month 3). According to protocol, ITT was performed including from the two other patients that only finished visit 3. The difference in FACT-G change from V1 to V5 between groups was not statistically significant (*P* = 0.755) (Fig. 3a).

**Fig. 4:**
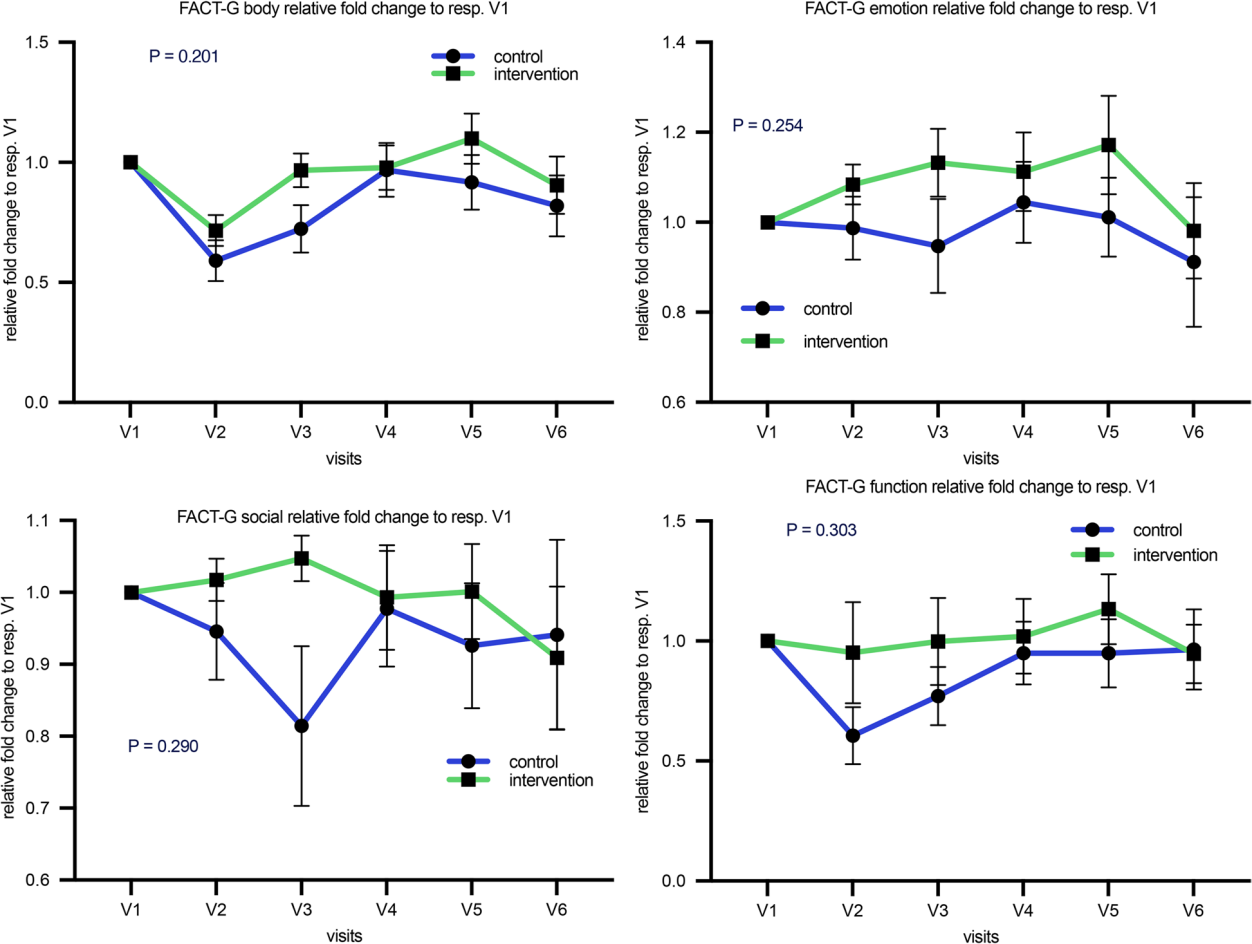
The mean fold change from baseline FACT-G in the different subcategories physical (**a**) l, emotional wellbeing (**b**), social wellbeing (**c**) as well as function in daily life (d). For visual purposes, error bars show the mean with SEM

FACT-G Sum Score over time shows a drop in QoL at the time of transplantation (visit 2) in both groups. The intervention group appeared to have a less severe decline and a trend toward recovery over time, but this difference was not statistically significant (*P* = 0.835 for group * visit interaction) (Fig. 3b). This pattern is seen also in the FACT-G subcategories of physical, emotional and social wellbeing as well as function (Fig. 4).

### HADS-D

**Fig. 5:**
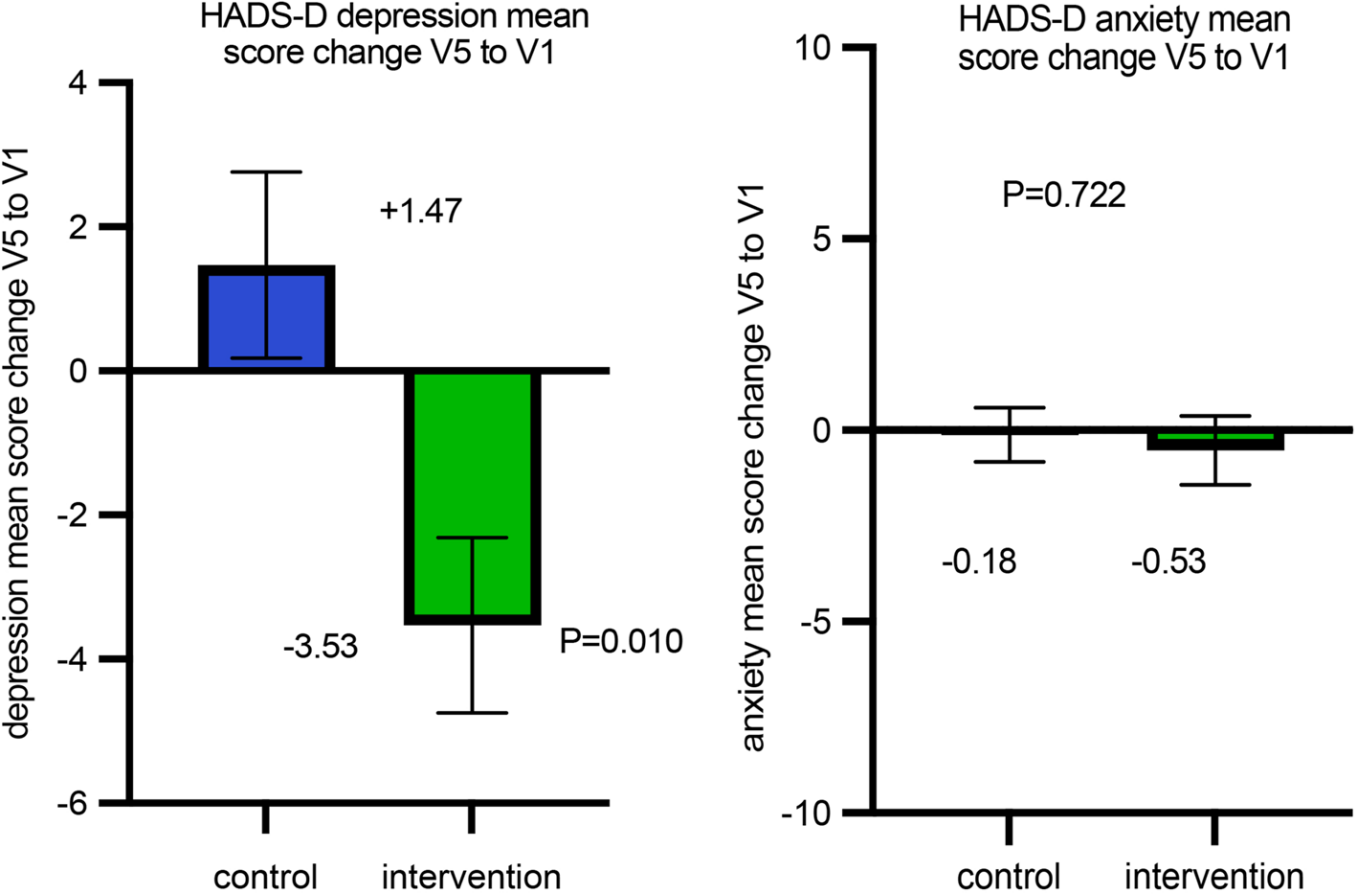
Development of depression (**a**) and anxiety (**b**) from V1 to V5 in control group and intervention group in the respective HADS-D score. The higher the number, the higher the anxiety/depression. For visual purposes, error bars show the mean with SEM

A total of 16 patients in the control group and 16 patients in the intervention group underwent visit 5 HADS-D assessments (month 3). According to protocol, ITT was performed including the two other patients that only finished visit 3. There was an increase in depression (mean + 1.47 points) in the control group V1 to V5 and a decrease (mean −3.5 points) in the intervention group (*P* = 0.010) (Fig. 5a). For anxiety, HADS-D score showed no relevant increase or decrease in either group at V5 compared to V1 with no difference between the groups (*P* = 0.722) (Fig. 5b).

### Acute GvHD

The intervention group developed aGvHD in 29.4% (5/17), while the control group developed aGvHD in 58.8% (10/17). Acute GvHD that needed systemic treatment (grade II and above) was observed in 17.7% (3/17) in the intervention group and 41.2% (7/17) in the control group. Moreover, the intervention group did not develop any severe aGvHD (defined as grade III or IV) vs. 17.7% (3/17) in the control group until visit 5 (suppl. mat. 2, table 1 ).

**Fig. 6:**
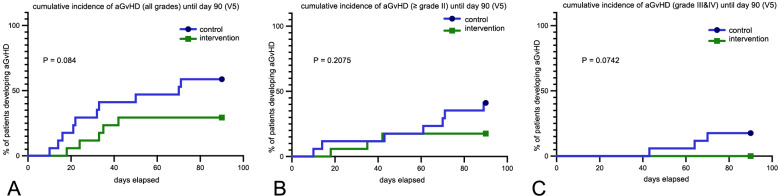
**a**, **b**, **c** Kaplan Meyer curve of aGvHD development until visit 5 (end of study treatment after 3 months) between the groups. From left to right: a = all grades, b = ≥ grade II, c = grade III&IV)

Kaplan Meyer analysis shows a trend in aGvHD reduction in the treatment group compared to the control group both after 3 months (visit 5, end of study treatment) (Fig. 6) and after 1 year (suppl. mat. 3 ).

### Progression Free Survival (PFS), Treatment Related Mortality (TRM) and Overall Survival (OS)

There was no difference in progression free survival (suppl. mat. 4, Figure 2a) or overall survival (suppl. mat. 4, Figure 2b) between the groups. Two patients in the control group experienced TRM compared to none in the treatment group (suppl. mat. 4, Figure 2c).

### Safety and complications of TCMA

Approx. 2000 acupuncture needles were inserted during the study period overall. There was no significant increase in occurrence of hematomas or soft tissue infections that needed treatment nor of positive blood cultures. A detailed description is found in the supplementary data (suppl. mat. 2, table 2).

### Other endpoints

In other endpoints, there was no significant difference between the groups (suppl. mat. 2, table 3). At the primary endpoint visit (V5), both mean ECOG-Performance Status Scale and mean Karnofsky Performance Status Scale did not show a significant difference between the groups (suppl. mat. 5).

## Discussion

Our study to our knowledge is the first to show that TMCA treatment is safe in the vulnerable group of patients undergoing aSCT. Our multimodal treatment was well accepted by study participants. It may reduce clinical depression. There is a trend mitigating the overall loss of Qol during the transplantation period and beyond, though the difference was not significant (*P* = 0.835 for group * visit interaction) (Fig. 3b). Furthermore, there was a trend to reducing aGvHD both in severity and in incidence.

Studies showing the safety of acupuncture in cancer-therapy related thrombocytopenia [11] and during cancer treatment [12] had led to more recent studies on even more vulnerable groups. In the study of Deng et al. [13], acupuncture was safe during autologous transplantation. Backed by these promising results on safety, our study is to our knowledge the first to show TCMA to be safe in patients undergoing aSCT. Despite the small number of participants, the number of approx. 2000 inserted needles total, each with a potential risk of bleeding or infectious complications, gives a more robust signal of safety. The low incidence of bleeding complications may also reflect our institution’s platelet transfusion threshold of 10,000/μL (or 20,000/μL for patients with fever or elevated bleeding risk), which ensured patients were not profoundly thrombocytopenic during acupuncture sessions. Particularly in the light of the growing evidence of the beneficial effects of acupuncture as a supportive therapy for a variety of symptoms during oncological treatments [14–17], a 15-fold increase in peer-reviewed publications in this field in the last 20 years [18] and current recommendations to integrate acupuncture into to routine clinical oncology practice both internationally [19] and nationally [20], our study may facilitate the conduct of more rigorous studies in this growing field. Caution advised translating this signal to other oncological patients, as transplant patients are more frequently being treated with systemic prophylactic or therapeutic anti-infective agents, which could mitigate potential infectious complications.

Depressive symptoms and reduced Qol occur often and may be severe during and after allogeneic transplantation. Although Qol measured by FACT-G did not reach statistical significance, our data show improvement of transplant related health issues on all subcategories and over the entire study period. The observed difference in FACT-G scores between groups (8.4 points, effect size 0.52) suggests a clinically meaningful improvement in QoL for the intervention group, per the FACT-G analysis manual, despite not reaching statistical significance, likely due to the small sample size. The depressive symptoms were reduced by this mixed-method approach using Clown-therapy, TaKeTiNa® music therapy and TCMA to a degree that would be clinically meaningful, if it can be reproduced in a larger patient sample.

Most studies using Clown-interventions have been conducted for pediatric cancer patients and have shown to improve psychological wellbeing [21–23]. Pilot studies with adult patients indicate similar effects [24]. The interventions usually focus on the passive role of the patient, “enjoying” the presence of the clown. Our approach, using the Metzler-Method® aimed to immerse the patient himself into roleplaying, thus facilitating the ability to act out feelings in a playful environment and being able to shift their perception of the present moment. A 73-year-old patient of the study recalled during an outpatient visit five years after transplantation: “When I see the others talking about how hard the transplantation was. I cannot understand it. For me it all went so fast and the roleplaying and music therapy helped me not to think about all that could happen”. Beside the subjective support it is to our knowledge the first published use of a Clown intervention in the setting of aSCT showing feasibility and safety.

Music therapy has shown to reduce side effects of autologous stem cell transplantation [25] and improve relevant health outcomes in cancer patients [26] and its routine implementation in cancer care is currently recommended by the national German society guidelines [27]. In addition, music therapy can also reduce psychological burdens such as depression in cancer patients [28] and during autologous stem cell transplantation [29]. There is evidence that these psychological burdens affect objective outcomes, even survival [6, 30–33]. In addition, they might even play a role for the development of aGvHD [34, 35]. Besides behavioral factors like medication adherence that might promote aGvHD in this group, there is also growing evidence of immunological changes that could contribute to this development [36, 37] and that could be changed by therapies that address depression.

It is to our knowledge the first published use of a music therapy intervention specifically in the setting of allogeneic SCT showing feasibility and safety in this particularly vulnerable group.

The role of TCMA in reducing depressive symptoms is still unclear due to the low quality of available studies [38]. There are however preliminary indications for its positive effect on Qol [39]. Our Focus using TCMA however was to reduce treatment related side effects (TRS) by using a state-of-the-art TCM diagnosis-based individualized Acupuncture at each visit. Among the TRS, particularly, aGvHD was of interest not only because of its clinical impact for the patients` morbidity and mortality, but because from the TCM point of view, it is a morphologically classical display of the imbalance of heat (Yang) and cold (Yin) that can be well addressed using TCM [10].

In this light, it is striking that regarding incidence and severity of aGvHD, there were on average 50% less events in the intervention group during the study period. The Due to the small patient numbers, a conclusion whether the lower incidence of aGvHD were random or due the intervention cannot be made. The aGvHD risk parameters were comparable overall (see Table 1). There were 2 GvH-locus mismatch donors that were female to male in the control group vs. 3 GvH-locus mismatch donors in the intervention group. There were 14 matched unrelated donors (MUD) and 1 matched related donor (MRD) in the control group vs. 8 MUD and 4 haploidentical donors and two MRD. The slightly reduced risk of developing aGvHD with haploidentical donors compared to MUD was not apparent in our study, as 3 out of 4 haploidentical transplantations developed aGvHD. In this light, it is striking that regarding incidence and severity of aGvHD, there were on average 50% less events in the intervention group during the study period. The development of aGvHD is complex. One key issue is the inability of the immunosuppressant agents or the reconstituted allogeneic immune system of the patient itself to regulate alloreactivity. All three therapeutic approaches of this study have a potential mechanism of action of moving the immune system to a more adaptive and regulatory state: TaKeTiNa music therapy and clown therapy could influence immune response through their change in psychological symptoms such as depression as shown above. TCMA has also shown to induce a variety of immunological changes [40] that could help the body to regulate and mitigate aGvHD.

### Limitations

Designing the study, we were aware that a study that uses a mixed-method intervention and is randomized but not placebo-controlled, yields both factors of bias that can influence the outcome of the study, as well as the limitations of attributing any meaningful effect to a specific intervention. A key limitation of this study is the failure to reach the planned sample size of 104 patients due to premature closure, resulting in only 36 patients enrolled. The small sample size likely reduced the statistical power to detect significant differences, particularly for QoL outcomes, and limits the generalizability of our findings. That we identified a significantly reduced rate of depression despite the early termination may suggest a stronger effect than anticipated when planning the study. Another limitation is the lack of blinding for outcome assessors. While participant blinding was infeasible due to the active nature of the interventions, blinding health workers or study personnel collecting questionnaires and clinical data could have reduced potential bias in outcome assessments. Future studies should consider assessor blinding to enhance rigor. The heterogeneity of the collective within the aSCT was another limiting factor. Unexpected or severe treatment related complications of the transplantation process or the recurrence of the disease early on can both shift questionnaire scores as well as other outcome parameters. To address the latter point, we included physical performance scores (ECOG, Karnofsky) to be able to correlate losses in quality of life due to only physical impairments such as severe infection or aGvHD, and analyzed the trend of Qol over time, to as best as we can reduce the influence of unexpected events in our discussion. We chose the mixed method to investigate the feasibility of the different approaches in this pilot study that had helped patients the most in our experience in our preliminary work. Patients were encouraged to perform TaKeTiNa and clown therapy exercises independently between sessions. But, the frequency and the extent of own practice was not systematically monitored and therefore not included in the effect calculations. It is noted that interpatient variability regarding self-exercises may have influenced outcomes and may therefore be a potential confounding factor that was not controlled for in this pilot study. Furthermore, the applicability of our findings may be limited by the availability of trained personnel to deliver TCMA, TaKeTiNa music therapy, and Metzler clown therapy. These interventions require specialized training and resources, which may not be readily available in all transplant centers. As a pilot study, this trial demonstrated TriCAM’s feasibility in aSCT, with high patient acceptance (95% interest rate) and no safety concerns. However, personnel changes and patient complications (e.g., severe infections or early relapse) disrupted continuity, contributed to early closure, and complicated follow-up—highlighting single-center vulnerabilities. Future trials could improve this through multi-center designs to share expertise, therapist cross-training for redundancy, and contingencies like backup staffing or hybrid models to ensure fidelity amid disruptions.

These studies could also explore scalable alternatives, such as simplified music or role-playing exercises, to enhance the feasibility of implementing complementary interventions in diverse settings. One of the methods, that was reported by patients to be particularly effective in relieving symptoms of depression was the TaKeTiNa-music therapy. Therefore, in a collaboration with the psychiatric department of the University Hospital of Erlangen, we have conducted a clean, randomized controlled trial to investigate it’s influence on patients with major depression (NCT05778643) [41] and we are currently designing an acupuncture study to investigate its effects on aGvHD.

## Conclusions

In summary, our study is the first one to show each of the therapies to be feasible and safe in the setting of aSCT in a randomized fashion. Because of its small patient size, firm conclusions about efficacy cannot be drawn. The promising results of this pilot study however give both a positive signal for safety and efficacy allowing the design. This is particularly important for acupuncture in highly immunocompromised patients with high risk of bleeding. Despite the small sample size, with over 2000 needle insertions, a strong safety signal can be drawn from this study for all oncological studies.

The preliminary positive impact on depression and trend toward improved QoL observed in this pilot study justify further investigation in larger, adequately powered trials to confirm the efficacy of TriCAM in aSCT patients.

## Supplementary Information

Supplementary Material 1.

Supplementary Material 2

Supplementary Material 3

Supplementary Material 4

Supplementary Material 5

## Data Availability

The datasets used and/or analysed during the current study are available from the corresponding author on reasonable request.

## Abbreviations

aGvHD: Acute Graft versus Host Disease
aSCT: Allogeneic Stem Cell Transplantation
ECOG: Eastern Cooperative Oncology Group (Performance Status Scale)
FACT-G: Functional Assessment of Cancer Therapy - General (German version)
HADS-D: Hospital Anxiety and Depression Score - German version
ITT: Intention-to-Treat
MRD: Matched Related Donor
MUD: Matched Unrelated Donor
OS: Overall Survival
PFS: Progression-Free Survival
PTSD: Posttraumatic Stress Disorder
Qol: Quality of Life
RCT: Randomized Controlled Trial
TCM: Traditional Chinese Medicine
TCMA: Traditional Chinese Medicine Acupuncture
TRM: Treatment-Related Mortality
TRS: Treatment-Related Side Effects
TriCAM: Complementary Medicine in Allogeneic Stem Cell Transplantation (study name)
WMWTest: Wilcoxon-Mann-Whitney Test
