# Supplementary material for: TriCAM (NCT02976558) – a randomized controlled pilot study of complementary medicine in allogeneic stem cell transplantation to improve quality of life
Source: BMC Complement Med Ther. 2025 Sep 8;25:326. doi: 10.1186/s12906-025-05058-8 (PMC12418651; doi:10.1186/s12906-025-05058-8)
Supplement: Supplementary file 1 — Supplementary Material 1. [file 12906_2025_5058_MOESM1_ESM.pdf]

TriCAM Übungsheft

TA KE TI NA

Rhythmus - Wahrnehmung - Stimmbildung - Körperbewusstsein

Was ist TaKeTiNa?

TaKeTiNa vermittelt Rhythmus, wie der Mensch ihn von Natur aus am besten erfassen und lernen kann: er führt direkt zur körperlichen Erfahrung rhythmischer Urbewegungen und zu den rhythmischen Grundbausteinen, auf der jede Musik der Welt aufbaut. Der Körper ist das Musikinstrument, die Begegnung mit Rhythmus ist daher entsprechend direkt und intensiv.

TaKeTiNa behilft sich der Stimme, den Händen und den Füßen. Es werden einfache Rhythmen, komplexe Rhythmen, oder repetitive Rhythmen benutzt um einen Zustand von Wachheit, Körperbewusstsein und spielerischer Freude zu schaffen.

In diesem Zustand wird unter anderem den natürlichen Prozessen und Emotionen die durch die Krankheit und Therapie entstehen können Raum gegeben diese zu beobachten und anzunehmen wie sie sind. Man muss weder fröhlich sein noch traurig sein. Man lernt die Melodien, den Rhythmus, den Raum und die Emotionen zu beobachten ohne sie zu beurteilen. Dadurch können sie sich ausdrücken und auflösen.

Wie sollte ich TaKeTiNa anwenden?

Einführung zu Beginn:
Ich werde Ihnen zu Beginn dieser Reise, auf die Sie sich begeben haben eine Anleitung geben, was TaKeTiNa ist und wie man es für sich nutzen kann. In den Einheiten werden wir Beispiele einüben, die Sie auch im Heft finden.

Übungen individuell gestaltbar:
Tägliche Übungen je nach individuellem Bedarf und dem aktuellen Zustand helfen Ihnen, die Krankheit besser zu verarbeiten, die körpereigenen Kräfte zu stärken und die Regulationsmechanismen des Körpers wieder zu aktivieren.

Welche Übung bei was anwenden?
Zu jeder Übung gibt es eine kurze Erklärung und Tipps, wann sie am förderlichsten für die Gesundheit sein kann.

Täglich 5-10 Minuten für beste Resultate
Ich bitte Sie täglich 5-10 Minuten einzuplanen, um eine oder mehrere der Übungen durchzuführen. Nur durch die konsequente Anwendung kommt es zu Resultaten.

TaKeTiNa Übung 1:
Richtig Atmen

Die meisten Menschen atmen sehr flach und schöpfen nicht annähernd ihre Lungenkapazität voll aus. Dadurch kommt es zu einer Minderbelüftung bestimmter Lungenanteile und damit zu einer erhöhten Anfälligkeit für Lungenentzündungen. Darüber hinaus kommt es zu einer Minderversorgung der Organe mit Sauerstoff. Aus Sicht der chinesischen Medizin verhindert die flache Atmung zudem den natürlichen Fluss der Körperenergien und verursacht Blockaden.

Um richtig atmen zu können und den Erfolg des richtigen Atmens zu erkennen, gibt es ein paar Hilfsmittel. Wichtig ist, dass Sie durch die Nase einatmen und durch den Mund ausatmen.

Luftballon
Stellen Sie sich vor, dass der Bauch ein entleerter Luftballon wäre. Ihre Luftröhre ist der Luftballon-Hals. Nun stellen sie sich vor, dass sobald Sie sich völlig entspannen und oben aufmachen, dass Luft in diesen Luftballon strömt und sich auffüllt. Wenn Sie bei diesem Bild bleiben muss sich beim Einatmen Ihr Bauch ausdehnen. Mit dem Ausatmen stellen Sie sich vor, dass der Ballon sich wieder leert und der Bauch wieder absinkt.
Ausdehnen
Als nächstes sollten Sie Ihre Hände in den Bereich der Nieren am Rücken kurz unter dem Rippenbogen legen. Versuchen Sie nun beim Einatmen zu spüren wie sich, so wie sich der Luftballon ausdehnt Ihr Rumpf im Bereich der Hände gegen Ihre Hände drückt. Es ist eine sehr effektive Methode Ihre Aufmerksamkeit auf die Bereiche Ihrer Lunge zu legen, die sie normalerweise nicht gebrauchen. Sie können den Atem immer tiefer in den Bauch kommen lassen und immer weiter gegen Ihre Hände ausdehnen lassen.
Fließen lassen
Sobald Ihnen diese 2 Elemente vertraut sind, lassen Sie Ihren Atem tief in den Bauch fließen und während Sie einatmen stellen Sie sich vor, dass Energie kurz Unterhalb des Bauchnabels nach oben entlang der Wirbelsäule bewegt und bis zum Kopf hoch geht und beim Ausatmen diese Energie wieder nach unten, aber auch in den ganzen Körper fließt. Stellen sie sich vor wie diese Kraft in die Hände und Füße fließt. Wenn Sie die Übung richtig anwenden werden Sie nach einer gewissen Zeit Wärme oder ein Kribbeln spüren

Wann? : Am besten täglich mehrmals

TCM: Die Übung verstärkt die Wirkung der Akupunktur, vermindert aus Sicht der chinesischen Medizin durch Förderung des Energieflusses Blockaden und führt somit

Schulmedizin:
Durch die bewusste Wahrnehmung des Atems und der tiefen, langsamen Ausführung reduziert diese Übung über Aktivierung des Parasympathikus körperlichen Stress und fördert die Regeneration des Organismus. Insbesondere können die bei der Transplantation häufigen Lungenentzündungen durch dieses Atemtraining reduziert werden. Fragen Sie Ihren Physiotherapeuten nach weiteren Übungen!

TaKeTiNa Übung 2:
Die Stimme aktivieren

Diese Übung knüpft an die erste an. Fließt der Atem nun mühelos und tief herein und heraus, können Sie beginnen beim Ausatmen einen Summton zu machen. Die Höhe des Tones darf sich bei jedem Mal ändern so wie es Ihnen gefällt. Nach ein paar Atemzügen öffnen Sie nun den Mund um einen Ton zu produzieren. Diesen Ton halten Sie bitte so lange wie Sie ausatmen.

Entspannung
Achten Sie bei jedem Ausatmen darauf dass der ganze Körper, insbesondere aber der Bauch, der Brustkorb, der Hals, die Schultern, die Zunge, die Stimmbänder und der Kiefer entspannt sind. Dadurch wird der Ton resonant.
Variation
Der Ton darf sich bei jedem Ausatmen in Lautstärke, Höhe und Druck ändern. Spielen Sie damit
Vibration
Bringen Sie nun mit der Stimme den gesamten Organismus zum Vibrieren. Achten Sie darauf und spielen Sie damit. Wenn Sie einen resonanten tiefen Ton singen, vibriert nicht nur der Brustkorb, sondern vielleicht auch der Bauch. Singen Sie einen höheren Ton, kann es sein, dass Ihre Nebenhöhlen vibrieren. Versuchen Sie tiefe und allmählich immer höhere Töne zu singen und lassen Sie den ganzen Organismus vibrieren.
Fließenlassen
Lassen Sie nun zusätzlich beim Singen die Energie durch den ganzen Körper Fließen.

Wann? : Am besten jeden Morgen nach dem Aufstehen

TCM: Die Übung verstärkt die Wirkung der Akupunktur und ist insbesondere für den Lungenfunktionskreis förderlich. Energetische Blockaden können durch die Vibration gelöst werden.

Schulmedizin:
Als Prophylaxe oder zur Therapie von Lungenentzündungen ist diese Therapie sehr gut geeignet, da durch die Vibration Schleim und Sekrete gelockert, gelöst und abtransportiert werden können. Auch bei Problemen mit den Nasennebenhöhlen ist diese Übung ideal.

TaKeTiNa Übung 3:
Der Puls und die Zwischenräume

Ein Klang, ein Wort, ein Ton - sie alle können nur dann entstehen wenn es dazwischen etwas gibt, das kein Ton, kein Klang, kein Wort ist. Musik, Rhythmus, Sprache, alle entstehen durch das Verhältnis von etwas und nichts. Auch in der Welt um uns herum geschehen Dinge, ist es still, herrscht rege Bewegung und Chaos, tiefe Stille und Frieden. Wir haben im 21. Jahrhundert gelernt, vor allem auf das “etwas” zu achten und weniger auf den Zwischenraum. Durch diese Übung wollen wir nicht nur das Bewusstsein für die Stille, den Zwischenraum wieder wecken, sondern zugleich auch die Verbindung mit unserem Körper stärken.

Den Puls fühlen: Legen Sie Zeige- und Mittelfinger einer Hand mit sanftem Druck auf die Innenseite des Handgelenkes unter dem Daumen. Hier sollten Sie ihren Puls fühlen können. Ist er schnell, langsam, kräftig, schwach, überflutend, zerfließend, hart, regelmäßig, unregelmäßig? Fühlen Sie ihn und versuchen Sie ihn innerlich zu beschreiben.

Auf den Puls sprechen: Beginnen Sie nun, während Sie Ihren Puls fühlen, genau auf dem Puls zu sprechen. Auf jeden Puls ein Wort. Achten Sie darauf wenn der Puls schneller oder langsamer wird und versuchen Sie immer auf dem Puls zu sprechen. Wenn Ihnen das gut gelingt, gehen wir zum nächsten Schritt über

Neben den Puls sprechen: Versuchen Sie nun in die Zwischenräume zu sprechen. Es folgt ein Puls, dann ein Zwischenraum in dem Sie ein Wort sprechen, dann wieder ein Puls. Es ist sehr ungewohnt und anfangs kann es sein, dass Sie schneller werden, oder sogar eine gewisse Nervosität sich aufbaut. Vielleicht zieht es Sie auch immer wieder auf den Puls. Beobachten Sie was passiert. Probieren Sie auch aus, im Zwischenraum zwischen den Pulsen immer noch das Wort “Pause” oder “Stille” zu sprechen und schauen Sie ob es Ihnen gelingt dass sich die Stille weiter und weiter ausweitet.

<u>Wann?</u>: Am besten immer dann, wenn Sie sich ängstlich oder nervös fühlen. Dadurch dass Sie genau auf dem Puls sprechen wird Ihnen erst einmal bewusst was für eine Qualität Ihr Puls hat. Sie haben Kontakt zu Ihrem Körper aufgenommen und können nun durch Ihre wache, fühlende Aufmerksamkeit seine Funktionen beeinflussen. Dadurch dass Sie dann neben dem Puls sprechen achten Sie mehr auf die Stille, was eine beruhigende Wirkung haben kann.

<u>Schulmedizin</u>: Bei Nervosität, Schuldgefühlen oder Zukunftsängsten. Diese Übung zieht Aufmerksamkeit vom Verstand ab und bringt Sie zum einen zurück in ihren Körper und damit in den jetzigen Augenblick, zum anderen aber auch in die Stille. Indem Sie auf die Stille achten, werden Sie still.

<u>TCM</u>: Über den Puls kann ein geübter Arzt der chinesischen Medizin den ganzen Körper erfahren. Durch Ihre Aufmerksamkeit auf den Puls setzen die Selbst-Regulationsmechanismen des Körpers wieder ein

TaKeTiNa Übung 4:
Rassel LaGa - Die gleichzeitige Wahrnehmung

Beginnen Sie die Übung indem sie gleichmäßig und resonant die Silben Ga Ma La fortlaufend und in gleichem Tempo singen.

Ga Ma La Ga Ma La Ga Ma La Ga Ma La

Beginnen Sie nun, während sie weitersprechen, das La mit der Stimme zu betonen. Als nächstes zeigen sie mit der linken Hand in den Raum, wenn sie das La sprechen. Während Sie das La weiter mit ihrer linken Hand zeigen, betonen sie das Ga das darauf folgt und führen Sie auch das in ein Zeigen mit der linken Hand über. Während Sie weiter sprechen führen Sie die Handbewegungen auf La nun in eine Außendrehung der Hand so dass die Handinnenfläche nach oben zeigt und auf Ga in eine Innendrehung der Hand so dass die Handinnenfläche nach unten zeigt. Nehmen Sie als nächstes die Rassel in die linke Hand und führen die Bewegung fortwährend sie die Silben sprechen. Sie können diese Schritte auch auslassen und direkt in die Bewegung übergehen, wenn es Ihnen leicht fällt. Auch ist es möglich die rechte statt die linke Hand zu nehmen.

Freies Tönen: Während Sie mit der linken Hand auf die Silben La Ga mit der Rassel spielen, fangen Sie an Übung 1 oder 2 durchzuführen. Lassen Sie Ihre Stimme erklingen und singen Sie lange Töne. Gehen Sie irgendwann in ein freies Singen über. Um zu gewährleisten, dass Sie noch im richtigen Rhythmus spielen empfiehlt sich ein Metronom oder eine Metronom-App auf dem Computer oder Smartphone.
Partnerübung: Geben Sie einem Angehörigen oder Bekannten der Sie besucht eine Rassel und machen Sie die Übung zusammen. Schauen Sie, dass sie beide im Rhythmus bleiben und die Silben sprechen. Jetzt fängt einer an frei über etwas zu erzählen, zum Beispiel wie sein Tag war, was er gefrühstückt hat, wie es ihm geht. Sie werden merken, dass Sie oft dann rausfallen wenn Sie über etwas sprechen, was mit Emotionen beladen ist. Der Grund ist, dass dadurch Ihre Aufmerksamkeit entweder nach innen zur Emotion geht oder nach oben in den Kopf und ins Denken. Das ist nicht schlimm, sondern ganz normal. Holen Sie sich wieder zurück, bauen Sie den Rhythmus auf und sprechen Sie weiter. Es darf auch Freude machen raus zu fallen.
Variation Partnerübung: Wenn sich beide schon wohl in dem Rhythmus fühlen kann die Übung erweitert werden, so dass der der zuhört nicht nur die Silben spricht sondern auf die Silbe Ma einen kleinen Klatscher setzt.

<u>Wann</u>? : Am besten wenn Sie Besuch kriegen und gleich zu Beginn. Machen Sie sich es zur Gewohnheit in dieser Art miteinander zu sprechen. Durch die Synchronisation kommt man auf eine authentische Herzensebene mit der anderen Person.

<u>TCM</u>: Gegenwartsbewusstsein und Körperwahrnehmung helfen Blockaden zu lösen.

<u>Schulmedizin:</u> Meditative Achtsamkeitsübungen wie diese helfen nachweislich der Krankheitsbewältigung und dem seelischen Wohlbefinden

TaKeTiNa Übung 5:
Theta Drumming

Theta-Drumming ist eine der ältesten medizinischen Anwendungen und hat sich auf der ganzen Welt in ähnlicher Form unabhängig voneinander entwickelt. Es scheint, als gäbe es eine Urkraft in uns, die mit dieser Art des Trommelns in Resonanz geht. Unter Theta-Drumming verstehen wir ein wiederkehrendes regelmäßiges Schlagen der Trommel mit einer Frequenz von 140-220 bpm (um 4 Hertz). Durch die repetitive Frequenz hat man zeigen können, dass auch die Gehirnwellen sich entsprechend anpassen und man vermehrt die sog. Theta-Wellen hat. Theta-Wellen produziert das Gehirn vor allem kurz vor dem Einschlafen, in der Zeit wo man sowohl wach ist als auch so entspannt, dass man fast schläft. In den meisten alten Kulturen der Welt, ob Asien, Europa oder Amerika wurden in der einen oder anderen Form gemeinsame Rituale mit dieser Art des Trommelns durchgeführt. Nachdem man ungefähr 5 Minuten in dieser Geschwindigkeit trommelt verursachen die nun vermehrten Theta-Wellen des Gehirns eine zunehmende tiefe Entspannung, während man aber gleichzeitig wach ist.

Theta-Drumming: Fangen Sie an etwa 3x pro Sekunde (sie können sich auch anfangs ein Metronom zu Hilfe nehmen) ganz leise auf die Trommel zu schlagen. Versuchen Sie gleichmäßig, sowohl von in der Geschwindigkeit als auch in der Stärke zu schlagen. Nach ein bis zwei Minuten können Sie nun lauter werden, bis sie die Resonanz der Trommel auch in ihrem Körper spüren.

Theta-Drumming Circle: Noch effektiver ist es, wenn Sie diese Übung mit jemandem zusammen, vielleicht sogar in einer kleinen Gruppe zu machen. Man sollte dann ebenfalls ganz leise anfangen und mit der Zeit lauter werden. Die Gruppe wird sich, sofern alle ganz aufmerksam bei der Sache bleiben und nicht in Gedanken abschweifen synchronisieren, das bedeutet, dass die Schläge immer präziser zusammenfallen. Das ist ein bekanntes Phänomen in der Musik, aber nicht nur da. Menschen, die zusammen spazieren gehen werden nach einer Weile ganz automatisch ihre Schritte synchronisieren. Menschen die sich gut verstehen werden ihre Bewegungen synchronisieren (gleichzeitiges unbewusstes Heben des Glases zum Trinken zum Beispiel). Die Synchronisation verbindet über den Rhythmus die Menschen und eine echte Beziehung kann sich entfalten.

Theta-Drumming und die Stimme: Während nun das Theta-Drumming das rhythmische Grundelement gibt dass konsistent weiterläuft, kann jetzt, wenn Sie möchten die Stimme dazu kommen. Fangen Sie, wie in der TaKeTiNa Übung 1 mit dem Atem an. Atmen Sie bewusst und tief, beobachten Sie Ihren Atem. Dann, ganz allmählich, stimmen Sie, so wie in der TaKeTiNa Übung 2. Irgendwann können Sie in ein freies Singen übergehen, ganz leise, ganz laut, ganz tief, ganz hoch, lassen sie los, entspannen Sie sich und lassen Sie zu dass Ihre Seele sich durch Sie ausdrückt.

<u>Wann</u>? Eine Zeit, in der Sie ungestört sein können. Fragen Sie nach, wann Sie mal 10 Minuten alleine sein können damit Sie nicht mitten drin gestört werden. Vielleicht möchte ein Familienmitglied, Freund oder Bekannter sogar mitmachen.

Der Clown und die Urgebärden der Beziehung

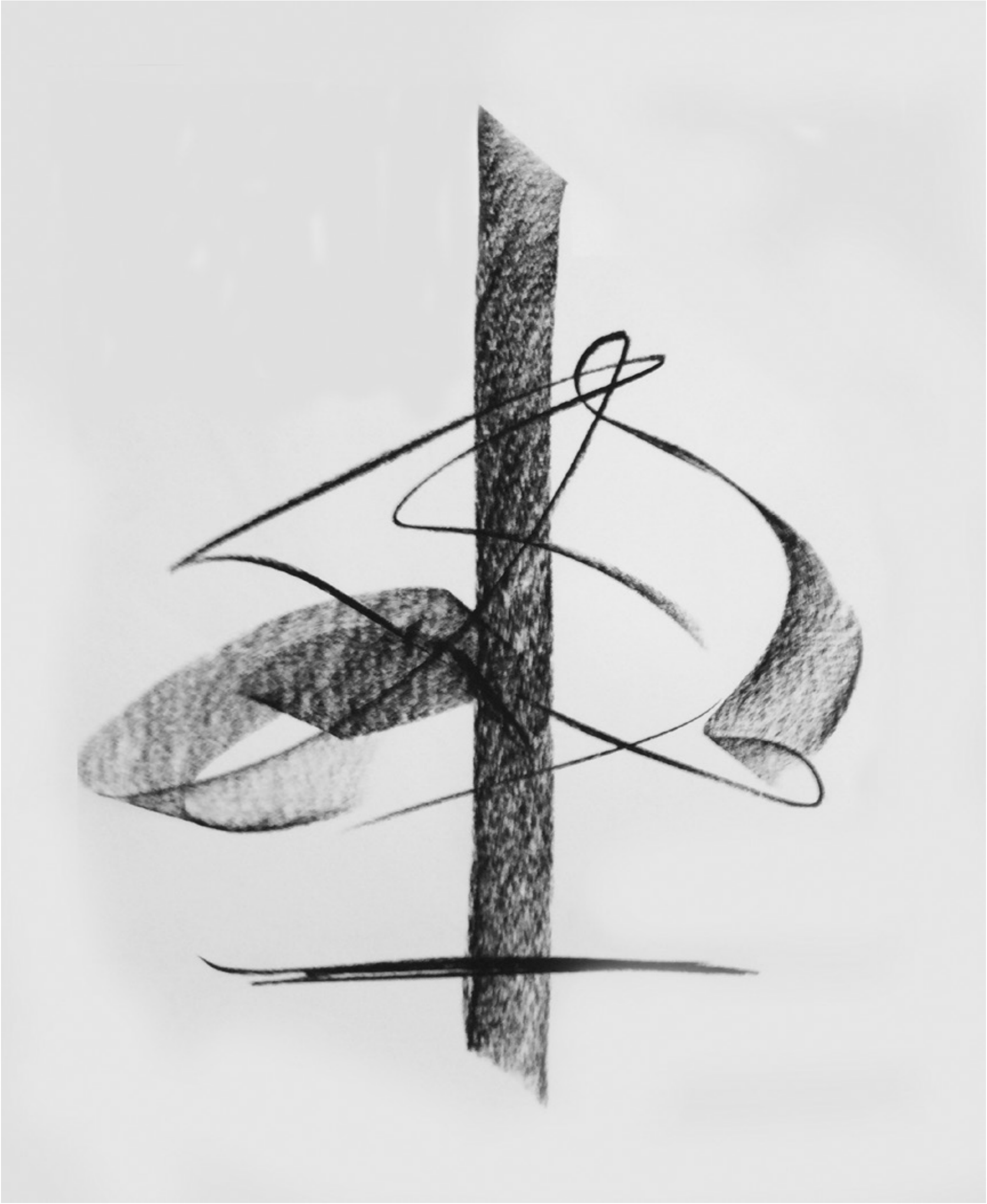

1. Einführung und die Regeln des Clowns

Was verstehen wir unter dem Clownspiel und die Gebärden?
Wenn man Clown hört assoziiert man damit in erster Linie Humor und das nicht ernst nehmen von Situationen. Dies kann gerade in einer so schwierigen Situation wie eine allogene Stammzelltransplantation unangebracht, ja sogar schädlich sein. Man assoziiert damit eine falsche Fröhlichkeit und das Herunterspielen von dem was gerade in einem vorgeht. Das ist nicht das, was wir unter dem Clown verstehen. Ich möchte Sie einladen Ihre Vorstellungen von Clown und seine Aufgaben für einen Moment loszulassen und lesen, was die Regeln des Clowns sind.

1. Nicht Wissen: Der Clown weiß nicht. Das Nicht-Wissen macht ihn offen zu erfahren. Er setzt sich aus

2. Nicht Spielen: Der Clown spielt echt. Er spielt in allem Ernst, was als sein persönliches Gefühl auftaucht. In diesem Sinne spielt er nicht

3. Immer Spielen: Aber er ist auch nicht privat. Was als sein persönliches Gefühl auftaucht, das macht er zum Spiel. Die Entwicklung des Humors schafft Loslösung und Abstand.

4.Fiasko: Der Clown scheitert. Er macht genau im richtigen Moment das Falsche. Er scheitert und gerät in Ausweglosigkeit, ins Fiasko

5.Insistieren: Der Clown gibt nie auf. Er insistiert. Im Konflikt, in der Panne, im Fiasko. Er insistiert. Er fängt immer wieder an.

6. Mit uns - Radar: Der Clown spielt im Kontakt mit dem Publikum. Er bleibt: mit uns. Im Radar.

7. Ankommen wollen und verbergen: Der Clown spielt was ihm peinlich ist und er verbergen will. Man sieht ihm an, wie er verbirgt.

8. Es sich entwickeln lassen: Es sich entwickeln lassen, wie Wasser alle Stellen ausfüllt bevor es weiterfließt. Der Clown ist möglichst immer absichtslos. Er ist direkt in die Situation eingebunden. So entwickelt sich das Spiel natürlich.

9. Den Keim einer Situation wahrnehmen: Den Keim einer Situation, einer Entwicklung wahrnehmen. Alles kann der Beginn einer großen Aufgabe oder eines großen Problems sein. Kein Augenblick und keine Handlung sind ihm zu gering.

10. Name - Der Clownspieler - Lachen / Lächeln: Jeder Clown hat seinen eigenen Namen. Der Clown kann nicht über sich lachen, aber der, der ihn spielt, muss über sich lächeln können, dann können die anderen Lachen. Das ist das Geheimnis des Clowns. Dass man über ihn lachen muss, weil er über sich lächeln kann. Der Clown macht es einem auch deswegen leicht - nicht nur, weil er überidentifiziert ist, sondern weil er gleichzeitig von naivem, tiefem Vertrauen ist. Er ist von Unverbundenheit beseelt. Darum kann er sich so verloren zeigen. Darum - während man über seine Verlorenheit lacht - wird man von seiner Ungebundenheit angesteckt

2. Die Grundgebärden (Urgebärden)

Jede Art der Beziehung zu einer Person, zu einem Gegenstand, ja sogar zu einer Situation kann auf sechs archetypischen Urbewegungen und deren Mischungen vereinfacht werden.
Durch diese Urbewegungen kann man bewusst dem Spielen Schattierungen und Charakter geben. Außerdem kann man sie in sich und anderen mit der Zeit auch im Alltag beobachten und mehr Bewusstsein über unbewusste Muster erkennen. Die Gebärden spiegeln auch innere Haltungen wieder, die einen in die Kraft bringen oder schwächen können.

Die Grundgebärden sind: **Deuten, Fließen, Wegschleudern, Innehalten, Rollen, Zu sich stehen.**
Auf den nächsten Seiten werden wir nun diese Grundgebärden näher betrachten.

Deuten - Der Bewegungscharakter der ersten Gebärde

Die erste Gebärde trifft wie ein Pfeil.

Vom Zielpunkt her ausholend bewegt sich die Gebärde auf direktem Weg, eine Gerade zeichnend, auf ihren Treffpunkt zu. In der Wende geschieht eine Sammlung und Ladung aufs Ziel hin.

Ich bin hier und das Ziel ist dort. Indem ich das Ziel erreichen will, wächst die Ladung, die sich aus dem Getrenntsein vom Ziel und dem Eins-Sein-Wollen mit dem Ziel befeuert.

Dieses Feuer reibt sich an diesem Gegensatz, sprengt die Sammlung in einem Zündungspunkt. Die Selbstbestimmtheit wirkt als Ich-Zündung. Wie ein Pfeil, schnell und einfach, bewegt sich die Gebärde und findet einen selbstbestimmten Endpunkt im Ziel. Angekommen im Ziel, wirkt das Ziel auf den zurück, von dem die Gebärde ausging und bestärktsein Stehen. Das Ziel ist Gegenstand und bestärkt den Eigenstand. Das Ziel gibt mir mich. Das Ziel gibt dem Ich Identität.

Die Gebärde beginnt direkt und endet direkt. So zeigt jemand unmittelbar auf ein Objekt, dem er gegenübersteht. Und im Sinne dieser willensbetonten Gebärde wird man etwas beginnen, in Angriff nehmen, selbstbestimmt gestalten, etwas erschaffen, sich auf ein Ziel zubewegen, sich ausrichten, für das eintreten, was einem gut tut, anführen, initiieren oder bestimmen, befehlen; oder sich entscheiden, sich durchsetzen, oder etwas nachjagen, etwas beanspruchen oder einen Tatbestand hinstellen und so fort.

In der egohaften Übertreibung dieser Gebärde kann man engstirnig seine Richtung verfolgen, andere überrennen und tyrannisierend seine Umgebung beherrschen. Im Vollzug dieser Gebärde entwickelt sich gegenständliches Bewusstsein.

Themen der ersten Gebärde sind:

Das Entdecken der Welt, Beginnen trotz Anfangswiderstand, Gestaltungskraft, Selbstbestimmung, freier Wille, Ziel- und Visionskraft, Unterscheidungskraft und Tatkraft und die Ausrichtung auf Werte.

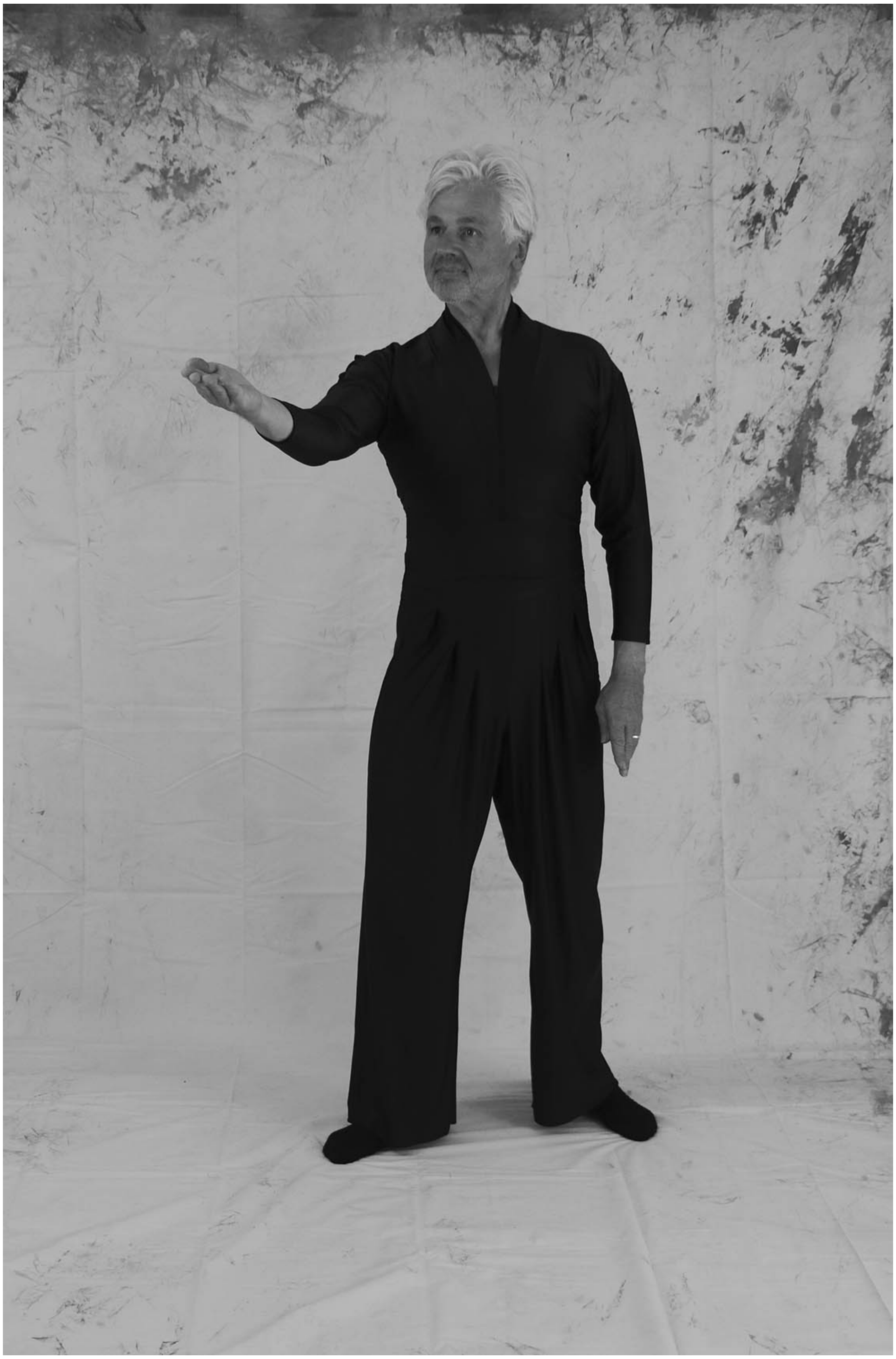

Fließen - Der Bewegungscharakter der zweiten Gebärde

Die zweite Gebärde fließt wie eine Welle.

Allmählich beginnt sie, wird sichtbar und vergeht. Sie hat schon begonnen, bevor man sie sieht und sie ist noch, nachdem sie im Sichtbaren zu Ende ist. Allmählich entsteht die Gebärde, ohne dass man einen exakten Anfang bestimmen könnte, allmählich schwillt sie an und wieder ab, Kurve um Kurve zeichnend in allen Variationen, um irgendwann allmählich wieder zu vergehen, ohne dass man ihr Ende exakt bestimmen könnte. Sie erwächst aus dem Nicht-Sichtbaren, wird dichter und wächst noch weiter, während bereits die Bewegung des Vergehens ihr entgegenkommt. Sie vergeht, wird weniger dicht und während sie noch ins Nicht-Sichtbare vergeht, kommt ihr bereits wieder die Bewegung des Werdens entgegen. Die beiden Prinzipien von Werden und Vergehen, von Aufblühen und Verwelken durchdringen sich. Die zweite Gebärde gehört mehr zu den Naturvorgängen als zu den Gestaltungskräften. Sie ist Ausdruck der Verbundenheit, der Ur-Verbundenheit, die immer schon da ist und die sich gerade in dieser Beziehung zeigt. So gibt jemand Raum, lässt Beziehung entstehen und vergehen, aufblühen und verwelken, ahmt nach, geht in Resonanz, lässt sich bewegen und beeindrucken. Mit der zweiten Gebärde lässt jemand einen anderen Menschen oder einen Eindruck an sich herankommen. So öffnet sich jemand dem, was ihm begegnet, taucht in einen Eindruck hinein und nimmt auf, was ist, und folgt einer Einladung oder Führung. Zum Beispiel breitet jemand seine Arme weit aus, um ein Gegenüber zu empfangen. Und im Sinne dieser warm fühlenden Gebärde wird man hinhorchen, anschauen, aufnehmen, berühren, oder sich zuneigen, sich einlassen, zustimmen, sich hingeben, sich sehnen, einladen, folgen, begleiten und so fort. In der egohaften Übertreibung der Gebärde kann man zu sehr an jemandem hängen, abhängig werden oder umgekehrt jemanden verführerisch an sich binden. Die zweite Gebärde wirkt über sich hinaus und verbindet. Indem jemand wahrnimmt und sich verbindet, stellt er sich in den anderen hinein- in seinen Stand- und kann von ihm aus schauen und mitfühlen mit dem, was ist. Mit der zweiten Gebärde entsteht „Inständliches Bewusstsein“.

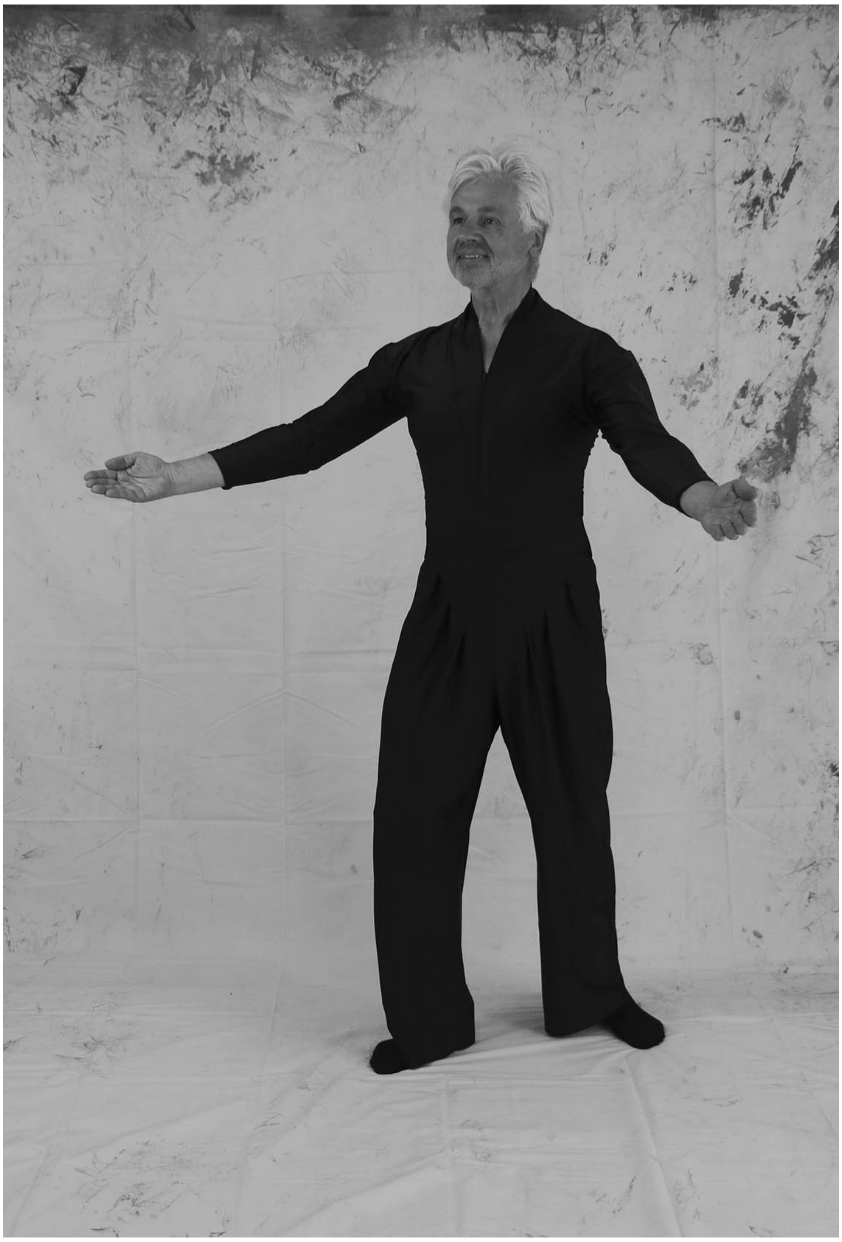

Themen der zweiten Gebärde sind:

Wahrnehmung, Nähe, Hingabe, Sympathie, Aufblühen und Verwelken, Entstehen und Vergehen, zuhören und aufnehmen, mitgehen oder sich bewegen lassen.

Wegschleudern -Der Bewegungscharakter der dritten Gebärde

Die dritte Gebärde wirft weg wie ein Wirbelwind.

Die Gebärde beginnt mit einem Ausholen, mit dem jemand an sich herankommen lässt, wogegen er sich abgrenzen will. Wie in einer Spirale nach innen, die sich in einem Innersten wendet und zu einer Spirale nach außen wird, schleudert sich ein Impuls von innen nach außen und setzt einen deutlichen Grenzpunkt. So erschafft jemand einen Abstand zwischen sich und etwas oder jemandem, so erschafft jemand Trennung. So schleudert jemand etwas von sich weg und bleibt übrig. Am Ende führt dieses Wegschleudern in eine stille Grenze und grenzt rundherum ab. Diese Grenze stehen lassend, ziehe ich mich von der Grenze zurück und indem ich mich zurückziehe, bleibt die Grenze in ihrer Wirkung bestehen. Von einem anderen Blickwinkel aus gesehen wirft oder schiebt jemand etwas von innen nach außen, drückt etwas nach außen, drückt etwas aus und „löst“ sich dadurch, zum Beispiel im sprechendem Ausdruck von einem Eindruck. Im Vollzug dieser Gebärde wird Ausdruck bewusst, und im Zusammenhang damit entwickelt sich abstandschaffende Bewusstseinskraft. Jemand wirft zum Beispiel, schwungvoll schleudernd etwas von sich weg, trennt sich von dem, was war oder ist, oder hält eindeutig ab, was kommen will, oder was ihm schaden könnte. Und im Sinne dieses zentrifugalen Impulses wird man in Distanz gehen, ablehnen, abwehren, sich weigern, verneinen, verbieten oder aufhören, sich verabschieden, hinter sich lassen, sich befreien, sich abstoßen, verwerfen. In der egohaften Übertreibung kann man verachten und vernichten.

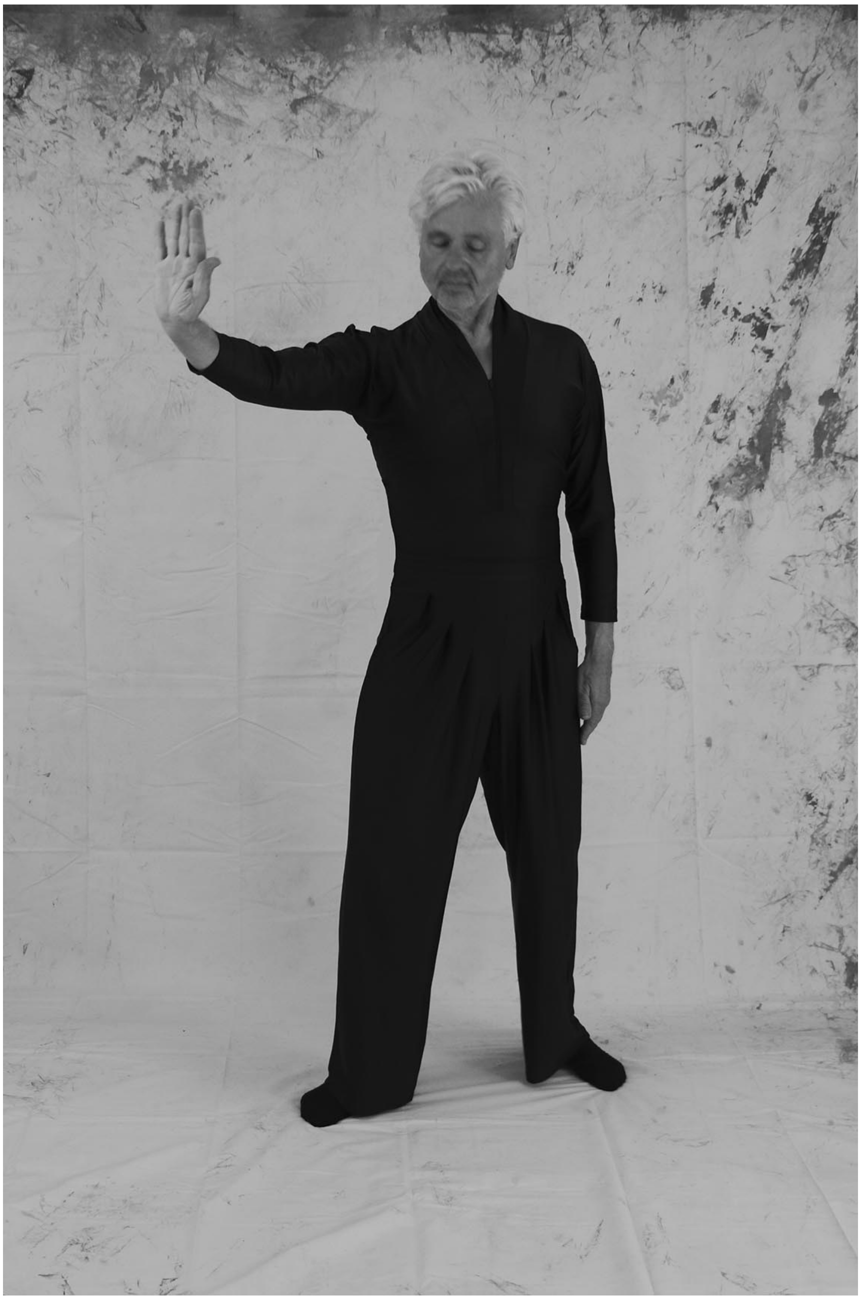

Themen der dritten Gebärde sind:

Distanz, Einzigartigkeit, Autonomie,
Begrenzung,
Antipathie.

Innehalten - Der Bewegungscharakter der vierten Gebärde

Die vierte Gebärde hält inne wie ein Stein. Im Innehalten nimmt jemand sich nach innen zurück. Sich selbst anhaltend und an sich selbst haltend, ist die Gebärde eine Nichtbewegung, ein Punkt, ein stiller Mittelpunkt. Als äußere Bewegung ist es die Bewegung, mit der jemand sich selbst berührt, um innezuhalten. Aus dem Eingebunden sein im Außen zieht er sich nach innen zurück. Er ist im Abstand. Das ermöglicht das Schauen, die Schau, die Betrachtung. Man kann auf dasjenige schauen, in dem man drinnen war. Man schaut aus Abstand, aus Ferne, aus Abstand zur Situation und auch aus Abstand zu sich selbst, aus Ich-Ferne. Man schaut aus Stille auf das, was sich bewegt. Man schaut aus Nicht-Bewegung auf Bewegung. Aus der Nicht-Bewegung, äußerlich oder innerlich, aus Bewegungsstille, Gedankenstille, Gefühlsstille, aus Ich-Stille schaut man auf das, was ist, was war oder was sein wird. Schaut jemand aus Stille, so hat er keine festgelegte, einseitige Sicht, keine Wertung, keine Einstellung. Vielmehr bezeugt jemand, was ist. Im Innehalten nimmt jemand sich zurück von dem, was war. Im Innehalten schaut jemand auf das, was sein wird. Jemand überlegt oder ahnt, was sein wird. Im Innehalten kann man erkennen. Das Innehalten kann dazu dienen, zwei Dinge gleichzeitig im Bewusstsein zu halten, zu vergleichen, zu bewerten und damit die Entscheidung vorzubereiten. Das Innehalten kann dazu dienen, einen Impuls zurückzuhalten, um dann vertieft auszudrücken, was man eigentlich ausdrücken will. Das Innehalten kann den Raum der Beziehung öffnen, die sich gerade vorbereitet. Jemand hat sich zum Beispiel auf sich selbst zurückgezogen, und aus dem Zustand distanzierter Geschlossenheit heraus betrachtet er, was ihn umgibt. Wenn ich zutiefst innen bin und auf mich schaue, dann ist es so, als würde ich wie von außen auf mich schauen. Da ist kein Hinwollen und kein Fortwollen. Da ist Bei-SichSein. Und im Sinne dieses Zustandes wird man sich sammeln und nachdenken, wird warten und zurückhaltend sein, geduldig sein, wird staunen und betroffen sein. In der egohaften Übertreibung kann man auch erstarren, sich aggressiv oder depressiv verschließen. So setzt sich jemand betrachtend auseinander mit dem, womit er im Ineinander eben noch verwoben war, stellt Eindrücke vor sich hin und hebt sie damit ins Bewusstsein. Im Vollzug dieser Gebärde entwickelt sich sachliches, zustandsbezogenes Bewusstsein, das Zeugenbewusstsein.

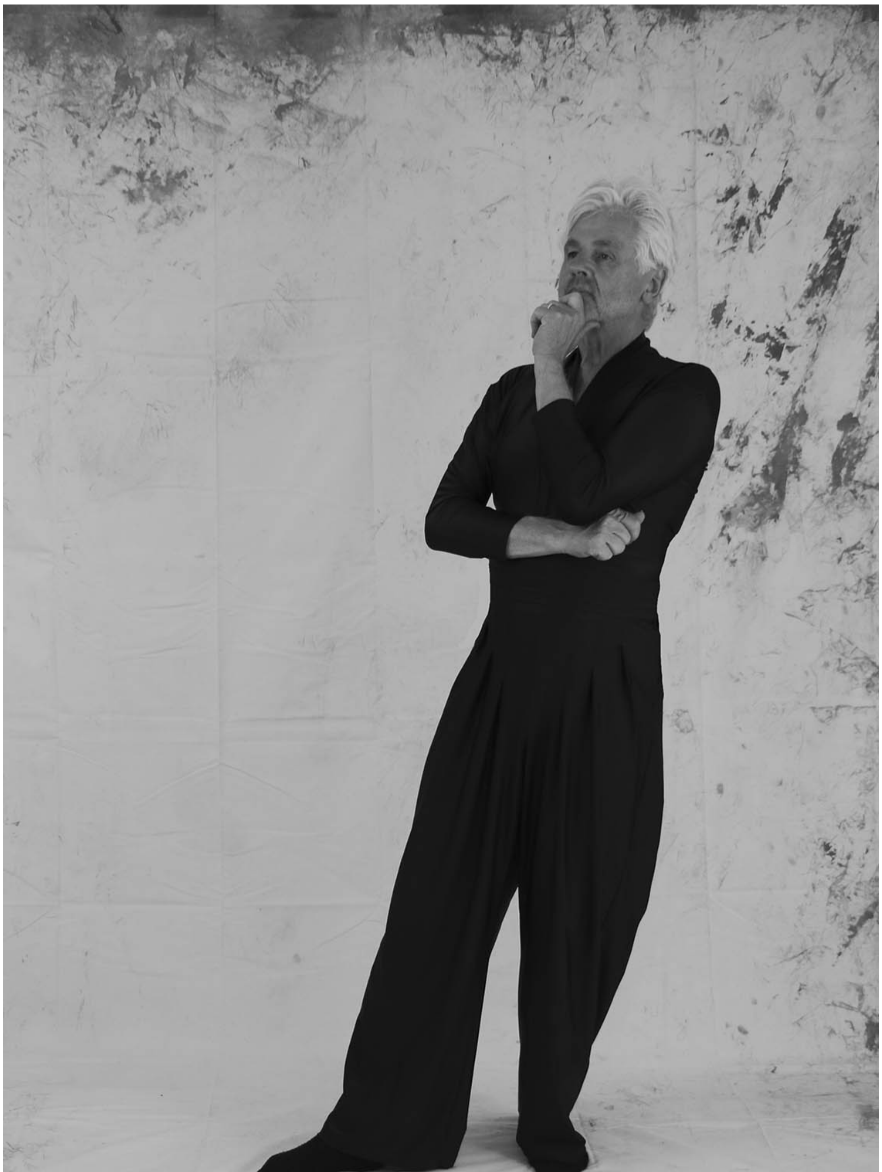

Themen der vierten Gebärde sind:

Betrachtung, Bezeugen, Schauen, Übersicht, Stille, Selbstwahrnehmung, aus Distanz auf die Situation oder die Beziehung schauen, Übersicht, Ferne, Nicht-Bewegung, Rückzug, Ausstieg, Zeitlosigkeit

Rollen - Der Bewegungscharakter der fünften Gebärde

Die fünfte Gebärde flackert auf und nieder wie eine Flamme, zuckt hin und her wie ein Blitz, rollt vor und rück wie ein Rad, oder vibriert wie eine gespannte Saite. Angetrieben oder gebremst von gegenpoligen Kräften wechselt die Gebärde in erregter Spannung von einer Richtung zur anderen. So lässt jemand das Fragen und Zweifeln ins Bewusstsein treten. Zum Beispiel zweifelt jemand im hierhin und dorthin Gezogen-Werden, zwischen diesem und jenem Anziehungs- oder Abstoßungspunkt. Oder jemand ringt um eine Sache, sei es gegen den Widerstand, der ihm entgegengesetzt wird, oder sei es gegen eine ihn zurückhaltende Tendenz. In Sinne dieser vibrierenden Gebärde wird man in erregter Spannung hin und her überlegen, wird zweifeln, schwanken, abwägen und probieren oder in Frage stellen, wird suchen, zögern, sich herantasten. In einer egohaften Übertreibung der Gebärde kann man auch misstrauisch und ängstlich sein, oder hysterisch umherirren und plötzlich den Kopf verlieren. Die fünfte Gebärde ist die Gebärde, mit der jemand sich in die Gegenpole hineinstellt, sich darin bewegt oder von den Polen bewegt wird. Wenn man sich die Peripherie eines Rades vorstellt, so ist in der Bewegung des Rades immer ein Vor und Zurück enthalten, ein Oben und Unten. In der fünften Gebärde zweifelt jemand, er ist in die Zwei eingespannt und im Hin und Her zwischen den Polen ringt jemand z.B. um eine Entscheidung. Er tastet sich wie gegen Widerstand nach vorn, probiert aus, experimentiert, erlaubt sich die Kraft unsicher zu sein und nicht zu wissen, wohin es geht, was Recht ist und was mit ihm übereinstimmt. Im Ringen darum, wie es gut weitergeht, geschieht Entwicklung. Man kann sich auch in den Konflikt hinein verlieren und sich hin und hergerissen fühlen, resigniert sein oder verwirrt nicht weiterwissen. Die fünfte Gebärde flackert hin und her, hoch und tief und in alle Richtungen wie ein Feuer. Wird das Hin und Her ganz schnell, dann wird die fünfte Gebärde zur Erregung, die einen sammeln oder zerstreuen kann. Die fünfte Gebärde ist die Bewegung, mit der man versucht, die Polaritäten des Lebens zu integrieren oder sich für eine Seite zu entscheiden oder in einen guten Wechsel zu gelangen.

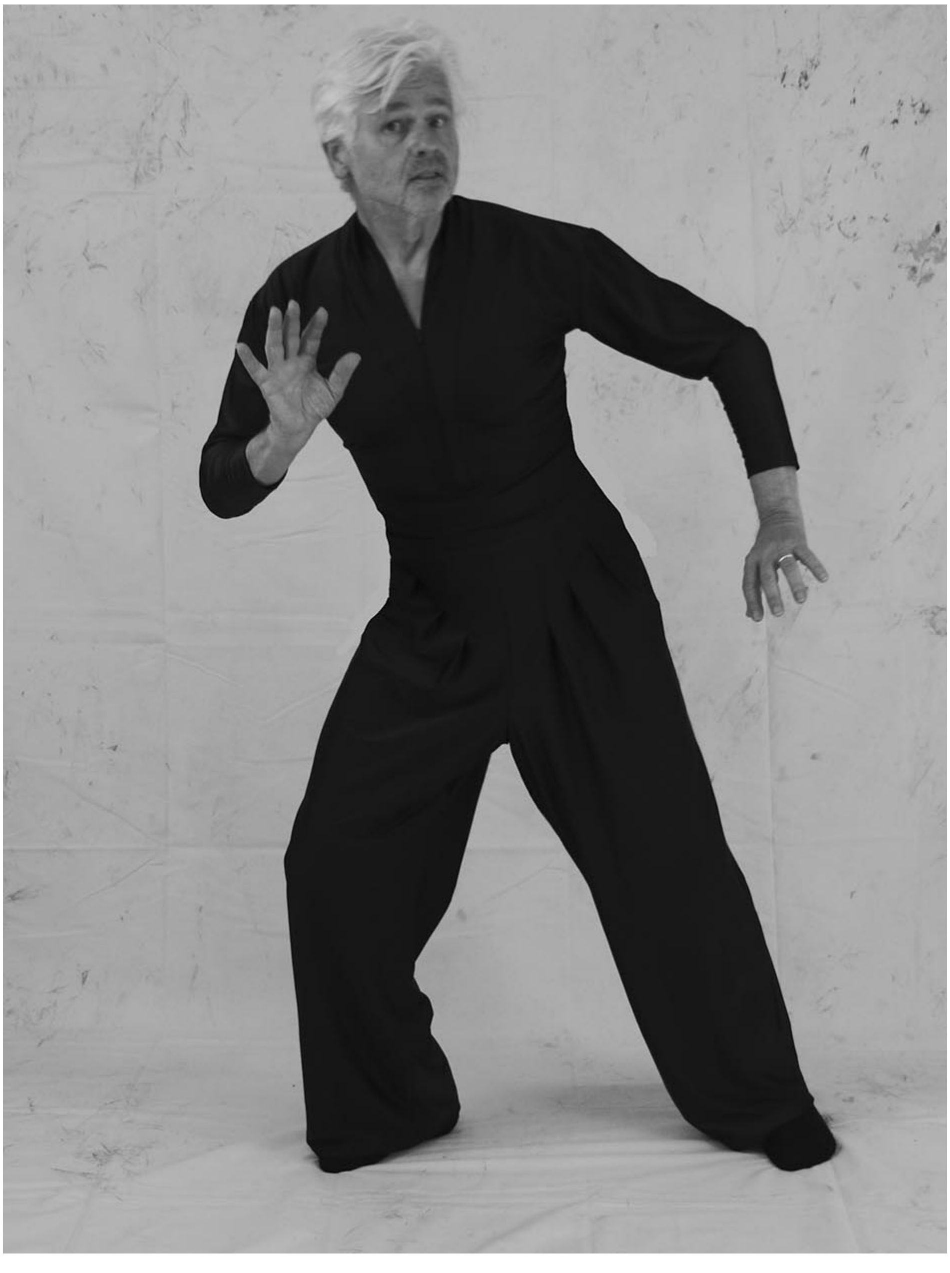

Themen der fünften Gebärde sind:

alle Gegenpol-Spannungen, alle Konflikt-Spannungen, die in der Begegnung der ersten vier Gebärden in Erscheinung treten können. Beispiele: Tun-Lassen, Beginnen-Abwarten, Nähe-Distanz, Wechsel-Dauer, Begrenzung-Unbegrenztes, Verbundenheit-Autonomie, Sympathie-Antipathie.

Zu sich stehen – Der Bewegungscharakter der sechsten Gebärde

Die sechste Gebärde steht wie ein Baum. Im Grund sich verwurzelnd und weit hinaufragend, zum Horizont sich ausspannend ruht die Bewegung in sich selbst. Diese Gebärde ist im Gebärdenspiel als einzige an eine äußere Form gebunden: Mit dem in der Weite ruhenden Blick schieben sich die Hände nah am Leib nach unten, als wollte man sich aus alter Haut herausschälen. So findet man diese Gebärde nicht als eine alltägliche Bewegung; sie ist nur Zeichen für einen inneren Akt und für eine Qualität, die sich aus diesem inneren Akt in alle Gebärden hineingeben kann. Ohne dass man diese Bewegung im Alltag findet, schwingt ihr Wesen in jedem Wort und in jeder Bewegung als Grundqualität mit. So schält sich jemand aus sich selbst heraus, so tritt jemand aus sich selbst hervor. Mit dem Vollzug dieser Gebärde erkennt oder erahnt jemand sein innerstes Sein und damit die Verbundenheit mit allem Dasein. Aus solchem Bewusstsein steht jemand zu sich selbst und zu allem, wie es ist. Im Sinne dieser Gebärde, zu der hin alles Gebärdenspiel strebt, wird man sich aus egohafter Abhängigkeit lösen, sich in Gegenwärtigkeit einer Situation stellen, etwas verantworten, sich verpflichten, aus tiefster Freiheit etwas auf sich nehmen.

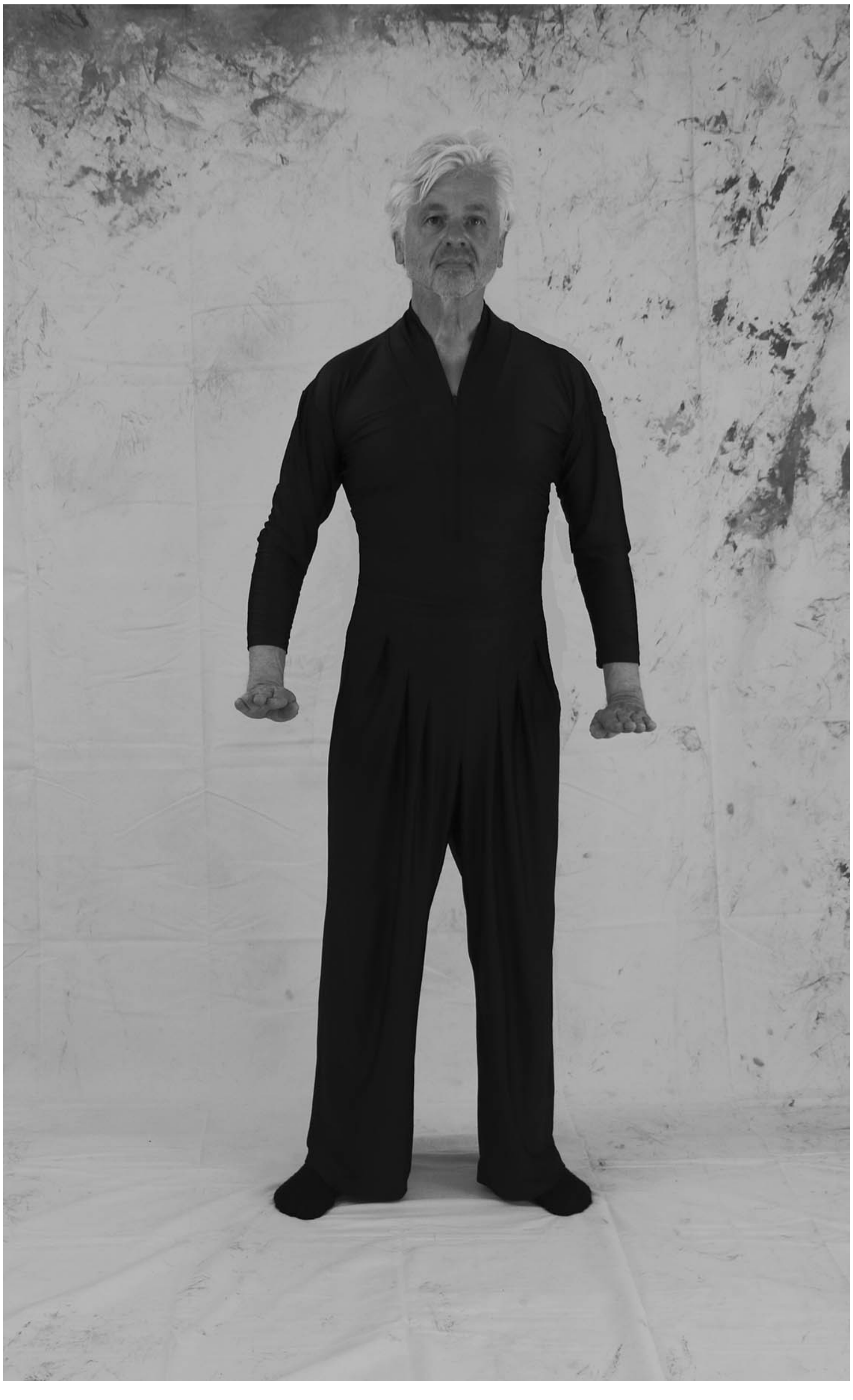

Themen der sechsten Gebärde sind: Anerkennen dessen, was ist, Selbstübereinstimmung, Antwortbereitschaft und Verantwortung, Verbundenheit und Losgelöstheit.

3. Die Kellerkinder

Neben den Urgebärden, mit denen jede Art von Beziehung ausgedrückt werden kann, gibt es noch Persönlichkeitszüge, die, wenn Sie im Gleichgewicht gelebt werden stärken können, im Überfluss jedoch die Schattenseiten bilden. Diese Schattenseiten sind dem Menschen der sie lebt oft unbewusst. Durch das Spielen dieser Schattenseiten, die wir Kellerkinder nennen, kann man einer Szene noch mehr Ausdruck verleihen. Während man sie spielt kann man in sich und anderen die einen oder anderen Kellerkinder wiederfinden, sehen ob man eher zu viel oder zu wenig dieser Qualität besitzt und eine wohlwollende Beziehung mit seinen eigenen Kellerkindern aufbauen und dadurch ihre innewohnende Kraft für sich nutzen. Diese potentielle Kraft eines jeden Kellerkindes steht jeweils rechts und ist mit einen * markiert.

Die Kellerkinder sind:

**Tranfunzel**	*** Intuition**
**Fetzer**	*** Tatkraft**
**Lästermaul**	*** Menschenkenntnis**
**Großkotz**	*** Weisheit**
**Flittchen**	*** Liebeslust**
**Geizhals**	*** Ordnungsliebe**
**Binnix**	*** Lebensfreude**
**Frau Wunderbar**	*** Urvertrauen**

Schatten kann man erleben, indem man eine bestimmte künstlerische Figur zu spielen und sich dazu in Beziehung zu setzt und sich fragt, was habe ich damit zu tun: ähnlich ich dem, was ich gespielt habe oder ist es eine Art Gegenteil von dem was ich eher bin und vielleicht vermeide. In der Schattenarbeit kann man sich fragen, wer nervt mich? Und es kann sein, dass mich der Neidbruder nervt: das ist die Qualität die mir fehlt und die mein Inneres mehr Aufträge zu entwickeln. Etwas davon zu entwickeln, ein gutes Maß das für mich stimmig ist. Es kann auch sein das der Schatten Bruder mich nervt. Das ist dann eine Qualität die mit der ich auch immer kämpfe und die ich ablehne in mir, weil ich etwas davon habe. Oder man sie kann sich fragen in welches Muster gerate ich immer wieder einmal. Man kann sich fragen wie bin ich als Kind angekommen und mache ich davon etwas heute noch? Man spielt ein Kellerkind und hat in gewisser Weise in dem man es ja nur spielt erst einmal damit gar nichts zu tun: das ist ganz unverfänglich. Dann kann man hin spüren und merken das man vielleicht so viel damit zu tun hat wie man im Moment fähig und bereit ist, es zu sehen. Es geht darum, alles was in einem als Schatten oder Lichtseite lebt anzuerkennen und mitleben zu lassen. Beim Schattenbruder ist es so: ich bin dem ähnlich der mich nervt. Ich ähnlich dem Franz, weil der ist auch ein Maulaufreißer. Wenn der kommt dann nervt er mich wahnsinnig, weil der erlaubt sich etwas, womit ich es schon immer schwer habe. Beim Neid Schatten ist es so: ich bin Sebastian, ich bin eher feinfühlig und sensibel. Und wenn der Fritz reinkommt, der nervt mich wahnsinnig. Weil - der erinnert mich an meinen Schatten. Mein Schatten ist das ich lernen könnte mich mir gemäß auszudrücken. Und das tue ich nicht, weil in meiner Feinfühligkeit und Sensibilität bleibe ich eher stumm und ausdrucksarm. Der Fritz ist wirklich ein Maulaufreißer. Er ist nicht positiv. Aber wenn ich von dem ein bisschen nähme, dann käme ich mir näher und würde mehr mich mir gemäß leben.
So tut mancher Schatten einem in einer gewissen Brise gut. In einem anderen Sinne könnte ich sehen - ich ähnliche diesem.
Manchmal ist es im Leben auch abwechselnd. Zeitweise ähnlich dem dann, gehe ich in das Gegen-Muster.

Clown - Übung 1:
Beziehung aufbauen

Diese Übung dient dazu, Bewusstsein für Gegenstände, Menschen und Situationen zu fördern. Wenn wir im Alltag etwas nehmen dann nehmen wir es, ohne vorher damit in Beziehung zu gehen und legen es mit genau so wenig Aufmerksamkeit wieder zurück, sobald der Gegenstand seinen Zweck erfüllt hat. Oft machen wir das unbewusst auch mit anderen Menschen. Bevor wir mit ihnen Beziehung aufbauen sprechen wir sie an oder berühren sie und überrumpeln sie dabei. Es ist auch möglich, dass sie das auch schon an sich durch andere Menschen gespürt haben. Diese Übung beschreibt das Grundprinzip, das, wenn es ein wenig eingeübt ist, auch auf alle Bereiche des Lebens übertragen werden kann.

Schauen Sie sich einen Gegenstand in Ihrer näheren Umgebung an, zum Beispiel ein Bleistift oder eine Gabel. Spüren Sie, wie Ihre Aufmerksamkeit auf sie ruht. Spüren sie den Raum, der zwischen Ihnen und diesem Gegenstand ist. Dieser Raum ermöglicht es, dass Sie diesen Gegenstand als getrennt wahrnehmen Jetzt wo Sie Beziehung zum Gegenstand aufgenommen haben greifen sie den Gegenstand in dem sie zunächst nur Ihren Arm soweit dahin strecken dass sie ihn berühren könnten um dann nach kurzem Innehalten den Gegenstand mit den Fingern zu greifen um dann schließlich nach einem weiteren kurzen Innehalten den Gegenstand aufzuheben. Realisieren sie, dass jetzt kein Raum mehr zwischen Ihnen und dem Gegenstand ist.
Rückwärtsbewegung: Führen sie den Gegenstand dahin zurück wo Sie ihn hinstellen möchten, lassen sie aber noch nicht los. Der Gegenstand berührt jetzt schon den Tisch, ist aber noch in Ihrer Hand. Vollziehen Sie nun bewusst eine Trennung mit dem Gegenstand und lassen Sie dann den Gegenstand mit Ihren Fingern los, halten Sie kurz inne und entfernen Sie nun auch den Arm vom Gegenstand. Halten Sie nun die Hand an Ihrem Körper und spüren Sie, dass Sie sich getrennt haben. Spüren Sie, dass wieder Raum zwischen dem Gegenstand und Ihnen entstanden ist.
Variation 1: Sie können auch mit anderen Menschen Beziehung aufbauen. Nehmen Sie den Menschen der vor Ihnen steht wahr. Mit einer innerlich ausholenden und dann noch vorne öffnenden Bewegung öffnen Sie sich dem Menschen etwas und sprechen erst dann die Worte. Wenn Sie die Beziehung beenden möchten, verabschieden Sie sich innerlich von der Person, machen eine Geste der Trennung (zum Beispiel die Handinnenflächen auf die eigene Brust und ein kurzes Nicken und vollziehen Sie dann die Trennung.
Variation 2: Wenn die Beziehung zu Gegenständen Ihnen leichtfällt und Sie schon spüren, wie Sie Beziehung aufbauen können, so können Sie auch andere Menschen am Aufbau der Beziehung und an Ihrer Trennung teil haben lassen. Gehen Sie mit einem Menschen der bei Ihnen im Zimmer ist in Beziehung, dann gehen Sie mit einem Gegenstand im Raum in Beziehung und folgen dem Ablauf wie oben beschrieben. Bevor Sie jedoch mit den Fingern den Gegenstand greifen, schauen Sie erneut zur Person, dann zum Gegenstand und greifen diesen mit den Fingern. Dadurch haben Sie die andere Person am Beziehungsaufbau teilhaben lassen.

Clown - Übung 2:
Gebärdendurchspiel - Heute ist mein Tag

In dieser Übung spielt man die Urgebärden 1-6 durch, wobei die Art wie man sie spielt durch das Thema: Heute ist mein Tag vorgegeben ist. Mein Tag im Sinne von, alles geschieht zu meinem Besten, ich habe Kraft, ich habe Vertrauen. Diese Übung hilft, auch in schwierigen Situationen die Ressourcen zu mobilisieren, die jeder in uns hat so dass wir mit Zuversicht und Optimismus in unserer Kraft stehen können und dadurch Dinge die nicht veränderbar sind annehmen können und Dinge die verändert werden können mit klaren Gedanken, Worten und Taten nach unseren Wünschen ändern können.

1. Dies ist mein Tag. Ich entscheide mich dafür diesen Tag zu leben, um ihn bewusst zu vollziehen, damit ich mich nicht unfreiwillig darin finde.

2. Ich nehme teil, an dem was geschieht, auch an Unvorhergesehenes. Ich lasse mich ein auf Beziehung.

3. Ich grenze mich ab gegen übergroße Erwartungen an diesen Tag und auch dagegen zu wenig zu erwarten. Ich muss nicht alles mitmachen. Ich kann auch nein sagen. Ich stehe Dir gegenüber. Ich achte Deine Andersartigkeit. Das Nein möge einen guten Platz in unserer Beziehung haben.

4. Ich lasse mich still werden und erlebe mich eingebunden darin, wo ich bin. Ich nehme mich selbst wahr. Ich schaue aus der Sammlung der Stille auf unsere Beziehung.

5. Ich stelle mich in Frage, in dem was ich tue und was werden will. Ich lasse mich hierhin und dorthin anziehen, es ist noch nichts entschieden, ich lasse mich in Frage stellen von dem, was mir begegnet, ich lasse den Zweifel zu, und ich ringe darum, aus dem Heute das Beste zu machen. Ich mache mich bereit, verschiedene Blickwinkel auf die Beziehung zuzulassen. Was willst Du von mir? Was brauche ich von Dir? Was will es mit uns?

6. Was auch immer geschieht, dies ist mein Tag Ich stehe zu der Beziehung, so wie sie jetzt ist und wird. Mit meinen bei mir sein bin ich bei Dir.

Clown - Übung 3:
Die Kellerkinder beobachten

Kellerkinder und Schattenarbeit
Die Kellerkinder sind ein System das acht grundsätzliche Schattenfiguren anspricht. Erkennt man eine seine Schattenseite nicht an, so verliert man die Ganzheit, die immer mehr dadurch entsteht, dass man Integration von Gegen Polen in sich zulässt. Die Clownsfiguren sind extrem gezeichnete Figuren der dunklen Qualitäten. Und es gibt entsprechend helle Qualitäten dazu. Die Clownfiguren sind erst einmal übertrieben. Aber es kann sein, dass man nur eine Brise oder eine Tendenz einer Figur hat. Bei der Tranfunzel beginnt das dort wo man zu viel geschehen lässt und nicht handelt wo eigentlich Handeln dran wäre (Fetzer)

Schatten kann man erleben, indem man eine bestimmte künstlerische Figur spielt und sich dazu in Beziehung zu setzt und sich fragt, was habe ich damit zu tun: ähnele ich dem, was ich gespielt habe oder ist es eine Art Gegenteil von dem was ich eher bin und vielleicht vermeide.

In der Schattenarbeit kann man sich fragen, wer nervt mich?

Und es kann sein, dass mich der **Neidbruder** nervt: das ist die Qualität die mir fehlt und die mein Inneres mehr Aufträge zu entwickeln. Etwas davon zu entwickeln, ein gutes Maß das für mich stimmig ist. Es kann auch sein das der **Schatten Bruder** mich nervt. Das ist dann eine Qualität die mit der ich auch immer kämpfe und die ich ablehne in mir, weil ich etwas davon habe. Oder man sie kann sich fragen in welches Muster gerate ich immer wieder einmal. Man kann sich fragen wie bin ich als Kind angekommen und mache ich davon etwas heute noch? Man spielt ein Kellerkind und hat in gewisser Weise in dem man es ja nur spielt erst einmal damit gar nichts zu tun: das ist ganz unverfänglich. Dann kann man hin spüren und merken das man vielleicht so viel damit zu tun hat wie man im Moment fähig und bereit ist, es zu sehen. **Es geht darum, alles was in einem als Schatten oder Lichtseite lebt anzuerkennen und mitleben zu lassen.** Beim Schattenbruder ist es so: ich bin dem ähnlich der mich nervt. Ich bin ähnlich dem Franz, weil der auch ein Maulaufreißer ist. Wenn der kommt dann nervt er mich wahnsinnig, weil er sich was erlaubt, womit ich es schon immer schwer hatte. Beim Neid Schatten ist es so: ich bin Sebastian, ich bin eher feinfühlig und sensibel. Und wenn der Fritz reinkommt, der nervt mich wahnsinnig. Weil - der erinnert mich an meinen Schatten. Mein Schatten ist das ich lernen könnte mich mir gemäß auszudrücken. Und das tue ich nicht, weil in meiner Feinfühligkeit und Sensibilität bleibe ich eher stumm und ausdrucksarm. Der Fritz ist wirklich ein Maulaufreißer. Er ist nicht positiv. Aber wenn ich von dem ein bisschen nähme, dann käme ich mir näher und würde mehr mich mir gemäß leben. So tut mancher Schatten einem in einer gewissen Brise gut. In einem anderen Sinne könnte ich sehen - ich ähnle diesem. Manchmal ist es im Leben auch abwechselnd. Zeitweise ähnele ich dem, dann gehe ich in das Gegen Muster.

Die Übung besteht darin sich am Morgen fest vorzunehmen auf eine Situation zu warten, in der der eine Person einen aufregt oder nervt. Sei es ein Arzt, ein Pfleger, ein Verwandter, die Putzfrau, ein Mensch im Fernsehen. Sobald das passiert schreibt man sich die Situation in einem Zweizeiler auf und, wenn man ein bisschen Abstand dazu gefunden hat (zum Beispiel ein bis zwei Stunden später), schaut man sich die Kellerkinder an und fragt sich, welche Qualitäten die Person hatte und welchem Kellerkind sie am ehesten entsprechen könnte. Spielerisch spielt man dann die Szene erneut mit den verschiedenen Kellerkindern und schaut, was in einem passiert.

Clown - Übung 4:
Die Clownsnase aufsetzen

Unser Verstand ist so beschaffen, dass wir alles kategorisieren und verstehen wollen. Einmal verstanden und eingeordnet, ist das Mysterium geklärt, die Frage ob eine Situation oder Person eine Gefahr birgt oder Freude bringt kann beantwortet werden. Und so neigt der Verstand höherer Lebewesen dazu den Tag und seine Umgebung so kalkulierbar wie möglich zu machen. So muss keine unnötige Energie verschwendet werden, um zum Beispiel seine Nahrung aufzusuchen. Diese Ökonomie, die unserem Organismus früher dem Überleben gedient hat nimmt in unserer modernen Zivilisation Ausmaße an, die krankmachen können. Bei vielen Menschen läuft jeder Tag gleich ab. Sie essen ähnliche Dinge, haben eine Routine in den meisten Sachen und haben alles, was unerwartet oder nicht kalkulierbar sein könnte weitgehend aus ihrem Leben eliminiert. Wenn man sie fragt wie ihre Woche war haben sie meistens nichts Besonderes zu berichten, wissen oft sogar nicht in wie weit sich verschiedene Tage voneinander unterschieden haben. Lebt man eine Weile so, so gewöhnt man sich daran, dass alles gleich abläuft und kalkulierbar ist so dass überraschende Vorfälle als Unwillkommen gesehen werden. Man beschwert sich, dass dies oder das anders gelaufen ist als man es sich gewünscht hätte. Mehr und mehr nimmt man Bewegung, Überraschung, Spontanität, Spielen und Begegnung aus seinem Leben. Und ehe man sich versieht wird das Leben immer grauer, eintöniger. Man ist gelangweilt, möchte aber nicht raus aus seiner gewohnten Haut. Viele Menschen verlieren mit den Aufgaben des Erwachsenseins zunehmend die kindliche, neugierige Freude sich neuen Situationen auszusetzen. Dadurch dass man nicht alleine ist, sondern die meisten Menschen um einen herum genau so leben verstärkt sich das Verhalten.

Die Clownsnase: Die Clownsnase ist ein Symbol. Es ist die kleinste Verkleidung. Es ist ein Symbol , dass man nicht weiß, was passieren wird. Dass man sich neuem aussetzt und wieder mehr Farbe in sein Leben bringt. Wenn man eine Clownsnase aufsetzt, egal ob man sehr traurig ist, Angst hat, wütend ist oder fröhlich ist. Etwas ändert sich. Man kann sich selbst nicht mehr ganz ernst nehmen. Man fühlt immer noch die Wut, die Trauer, die Angst. Man spürt sie, man versucht sie nicht zu überspielen, und dennoch schafft man Raum zwischen der Emotion und sich. Aber es verändert sich nicht nur die Beziehung zu sich selbst, sondern auch die Beziehung zu den anderen. Wenn jetzt eine Person in Ihre Gegenwart kommt und die Nase sieht, wird sie anders reagieren als die vielen anderen Male davor. Man weiß nicht was passieren wird, ob sich ein inspirierendes Gespräch daraus ergibt oder man die andere Person auf eine andere Weise kennen lernt. So bringt man allmählich wieder das Unvorhersehbare und Spontane wieder in sein Leben.

<u>Die Übung</u>: Setzen Sie die Clownsnase 5 Minuten täglich auf. Egal ob es beim Lesen, beim Spazierengehen, beim Zähneputzen oder in einem Café ist. Setzen Sie sie einfach auf, lassen Sie das, was gerade in der Situation passiert so sein wie es ist. Wenn sorgevolle Gedanken da sind oder Angst, beobachten Sie die sorgevollen Gedanken und die Angst und seien Sie sich gleichzeitig der Nase auf der Nase bewusst. Ganz ohne Urteil, ganz ohne etwas ändern zu wollen. Setzen Sie sie auf bevor sie etwas beim Bäcker bestellen, setzen Sie sich mehr und mehr aus und lassen Sie wieder das kindliche Leben zurückkehren, dass Sie einst so liebten. An den Reaktionen Ihrer Mitmenschen werden sie sehen, wie viel Freude Sie anderen Menschen damit bringen. Ein kleiner Moment, 5 Minuten und lächelnd geht jemand den ganzen Tag mit der Erinnerung durch den Tag, dass jemand mit Clownsnase Brötchen bestellt hat.

.

Clown - Übung 5:
Das Leben in Gebärden spielen

Diese Übung ist hervorragend dazu geeignet aus dem “Hamsterrad” auszutreten, in dem wir uns oft befinden. Man spielt sein Leben in der 1-3. und der 5. Gebärde. Man spielt seinen Alltag auf der Arbeit,, zu Hause oder in der Klinik. Es ist ratsam Alltagssituationen zu nehmen, die einem normalerweise Unbehagen bereiten oder die man nicht gerne macht. Zum Beispiel der stressige Job. Dann spielt man diese Situation mit einer oder allen der oben genannten Gebärden in übertriebener Form, geht richtig hinein, ist sozusagen voll im “Hamsterrad”. Immer wieder, zum Beispiel nach 30 Sekunden, geht man einen Schritt zurück und geht in die 4. Gebärde. Man wird plötzlich zum Beobachter und statt voll drin zu sein, schaut man jetzt von außen auf sein Leben das vor einem läuft. Das gibt Raum, Raum für Erkenntnis, Raum für Entspannung, Raum für Mut, Dinge zu seinem Besten zu ändern. Mit einer 6. Gebärde sagt man “ja” zu dem was gerade ist und geht wieder ins Spielen hinein. Das macht man am besten 3-4x. Während man rein und raus geht kann es sein dass man bestimmte Dinge erkennt. Vielleicht erkennt man dass die Situation auch weiter funktioniert wenn man etwas langsamer agiert oder eine Alltagssituation deswegen so lästig oder unangenehm ist, weil ein zu Viel der einen oder anderen Gebärde enthalten ist. Vielleicht braucht es die durchschlagende Entscheidungs- und Handlungskraft der ersten Gebärde, vielleicht doch mehr von der weichen, fließenden und harmonischen Kraft der zweiten Gebärde usw. Am Ende der Übung geht man ein letztes Mal einen Schritt zurück, geht in eine 4. Gebärde und schließt mit einer 6. Gebärde ab.

Spielen Sie diesen Durchgang 5 Minuten in unregelmäßigen Abständen, etwa 1x alle 1-2 Wochen. Sie hilft mit Abstand auf sein Leben und die Dinge zu blicken, die einen im Alltag am meisten beschäftigen und gibt dadurch Lösungsansätze. Man erfährt direkt und unvermittelt, wieviel von was einem guttut und was nicht.

Dokumentation der täglichen Übungen für mehr Bewusstsein und Gesundheit

Tag 1 ______________________   Tag 30 ________________________

Tag	TaKeTiNa	Clown	Tag	TaKeTiNa	Clown
1	☐	☐	31	☐	☐
2	☐	☐	32	☐	☐
3	☐	☐	33	☐	☐
4	☐	☐	34	☐	☐
5	☐	☐	35	☐	☐
6	☐	☐	36	☐	☐
7	☐	☐	37	☐	☐
8	☐	☐	38	☐	☐
9	☐	☐	39	☐	☐
10	☐	☐	40	☐	☐
11	☐	☐	41	☐	☐
12	☐	☐	42	☐	☐
13	☐	☐	43	☐	☐
14	☐	☐	44	☐	☐
15	☐	☐	45	☐	☐
16	☐	☐	46	☐	☐
17	☐	☐	47	☐	☐
18	☐	☐	48	☐	☐
19	☐	☐	49	☐	☐
20	☐	☐	50	☐	☐
21	☐	☐	51	☐	☐
22	☐	☐	52	☐	☐
23	☐	☐	53	☐	☐
24	☐	☐	54	☐	☐
25	☐	☐	55	☐	☐
26	☐	☐	56	☐	☐
27	☐	☐	57	☐	☐
28	☐	☐	58	☐	☐
29	☐	☐	59	☐	☐
30	☐	☐	60	☐	☐

Dokumentation der täglichen Übungen für mehr Bewusstsein und Gesundheit

Tag 60 ______________________ Tag 90 ________________ Tag 150 ____________________

Tag	TaKeTiNa	Clown	Tag.	T.	Cl.	Tag	T.	Cl.
61	☐	☐	91	☐	☐	121	☐	☐
62	☐	☐	92	☐	☐	122	☐	☐
63	☐	☐	93	☐	☐	123	☐	☐
64	☐	☐	94	☐	☐	124	☐	☐
65	☐	☐	95	☐	☐	125	☐	☐
66	☐	☐	96	☐	☐	126	☐	☐
67	☐	☐	97	☐	☐	127	☐	☐
68	☐	☐	98	☐	☐	128	☐	☐
69	☐	☐	99	☐	☐	129	☐	☐
70	☐	☐	100	☐	☐	130	☐	☐
71	☐	☐	101	☐	☐	131	☐	☐
72	☐	☐	102	☐	☐	132	☐	☐
73	☐	☐	103	☐	☐	133	☐	☐
74	☐	☐	104	☐	☐	134	☐	☐
75	☐	☐	105	☐	☐	135	☐	☐
76	☐	☐	106	☐	☐	136	☐	☐
77	☐	☐	107	☐	☐	137	☐	☐
78	☐	☐	108	☐	☐	138	☐	☐
79	☐	☐	109	☐	☐	139	☐	☐
80	☐	☐	110	☐	☐	140	☐	☐
81	☐	☐	111	☐	☐	141	☐	☐
82	☐	☐	112	☐	☐	142	☐	☐
83	☐	☐	113	☐	☐	143	☐	☐
84	☐	☐	114	☐	☐	144	☐	☐
85	☐	☐	115	☐	☐	145	☐	☐
86	☐	☐	116	☐	☐	146	☐	☐
87	☐	☐	117	☐	☐	147	☐	☐
88	☐	☐	118	☐	☐	148	☐	☐
89	☐	☐	119	☐	☐	149	☐	☐
90	☐	☐	120	☐	☐	150	☐	☐
