# Supplementary figures and images for: TriCAM (NCT02976558) – a randomized controlled pilot study of complementary medicine in allogeneic stem cell transplantation to improve quality of life
Source: BMC Complement Med Ther. 2025 Sep 8;25:326. doi: 10.1186/s12906-025-05058-8 (PMC12418651; doi:10.1186/s12906-025-05058-8)

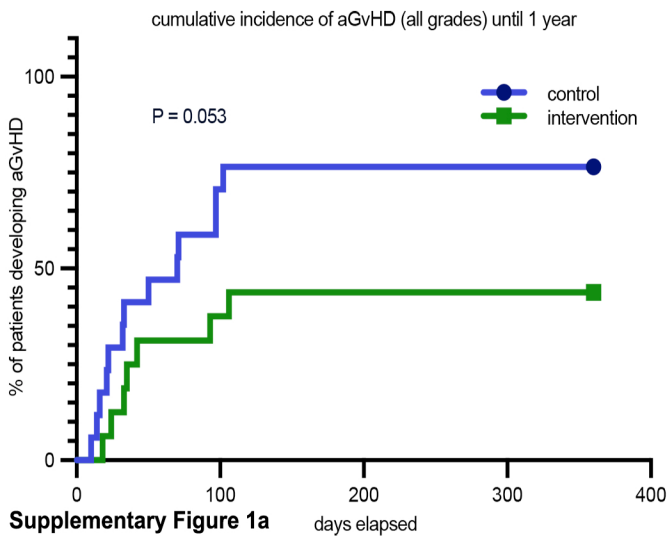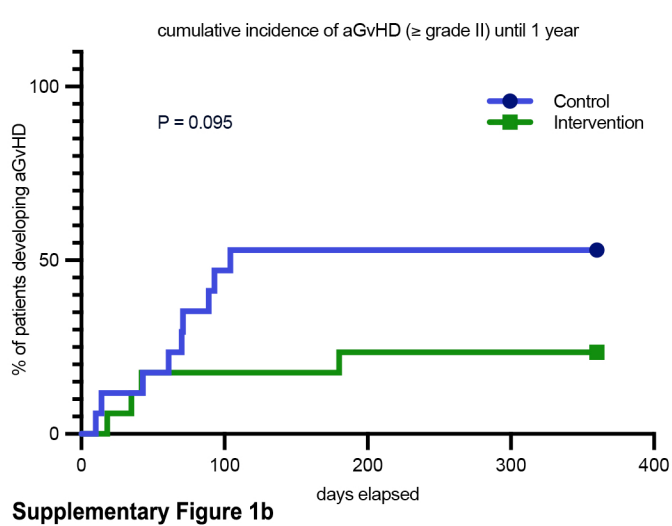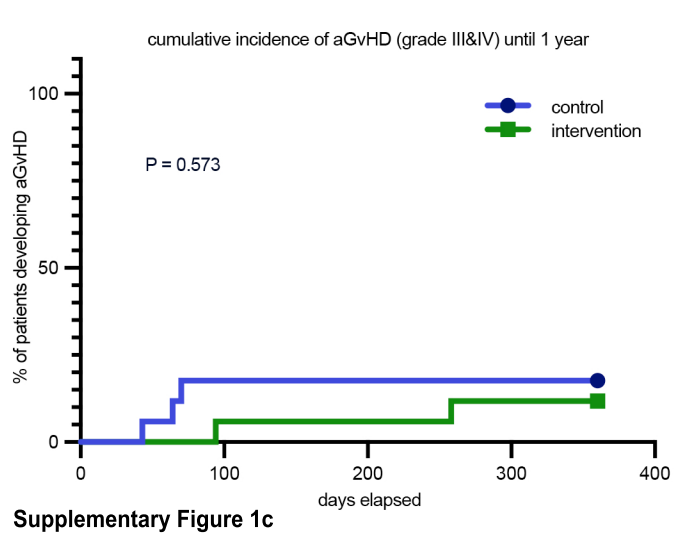

Supplement: Supplementary file 3 — Supplementary Material 3 [file 12906_2025_5058_MOESM3_ESM.pdf]
