# Supplementary material for: TriCAM (NCT02976558) – a randomized controlled pilot study of complementary medicine in allogeneic stem cell transplantation to improve quality of life
Source: BMC Complement Med Ther. 2025 Sep 8;25:326. doi: 10.1186/s12906-025-05058-8 (PMC12418651; doi:10.1186/s12906-025-05058-8)
Supplement: Supplementary file 4 — Supplementary Material 4 [file 12906_2025_5058_MOESM4_ESM.pdf]

## Supplementary Figure 2a

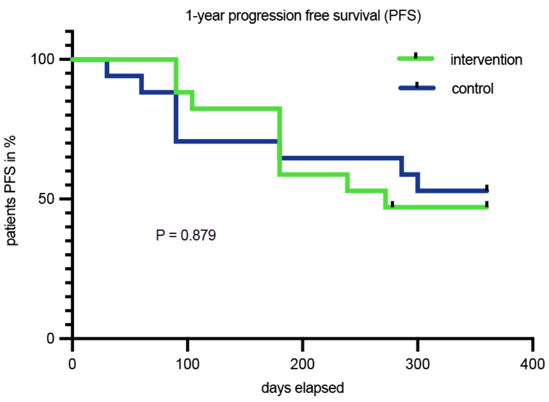


## Supplementary Figure 2b

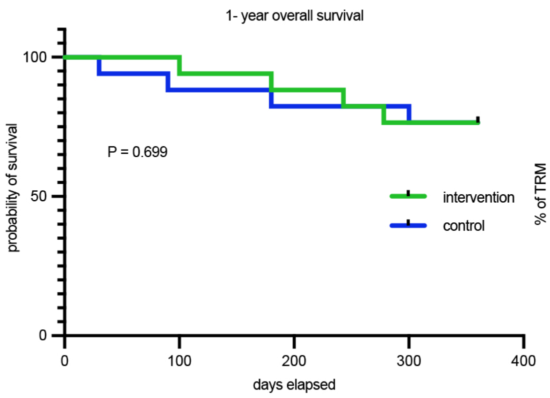


## Supplementary Figure 2c

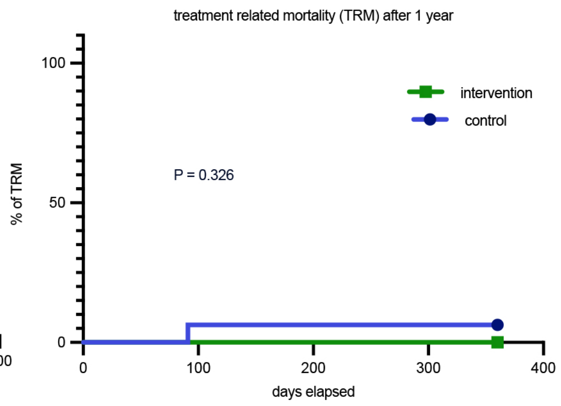


Supplementary figure 2a,b,c: from left to right: Kaplan Meyer curve of progression free survival until 1 year between the groups (2a), 1-year overall survival (2b) and treatment related mortality (TRM) after 1 year (2c).
