# Supplementary material for: TriCAM (NCT02976558) – a randomized controlled pilot study of complementary medicine in allogeneic stem cell transplantation to improve quality of life
Source: BMC Complement Med Ther. 2025 Sep 8;25:326. doi: 10.1186/s12906-025-05058-8 (PMC12418651; doi:10.1186/s12906-025-05058-8)
Supplement: Supplementary file 5 — Supplementary Material 5 [file 12906_2025_5058_MOESM5_ESM.pdf]

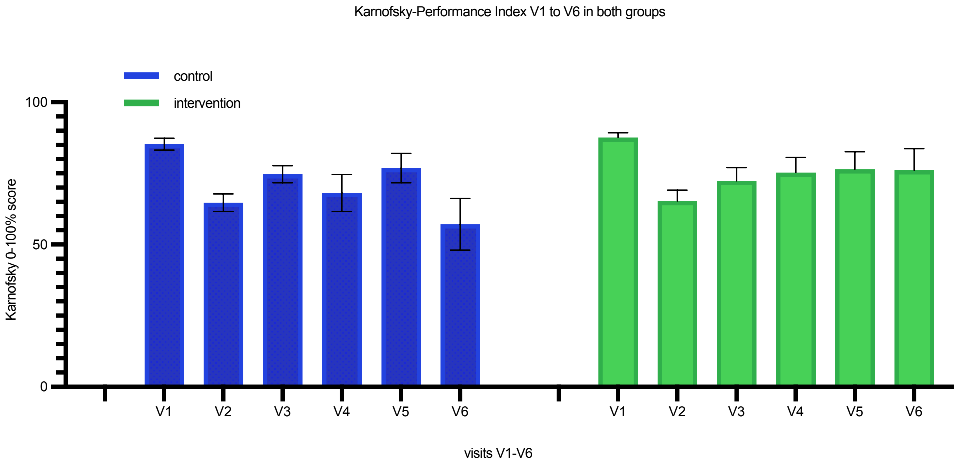

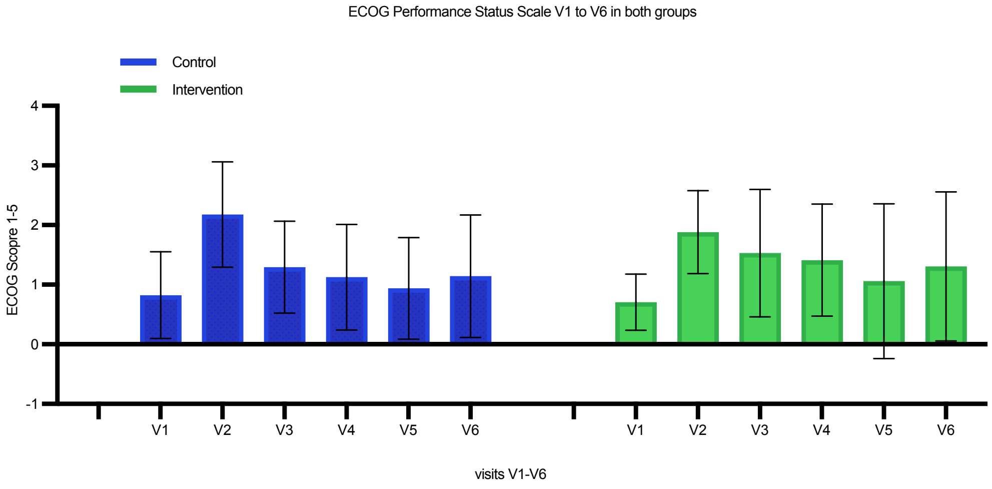

supplementary figure 3: The left graph shows the mean Karnofsky Performance Status Scale and the right graph the ECOG-Performance Status Scale development over time in the different groups. For visual purposes, error bars show the mean with SEM.
